# ih-RIDME: a pulse EPR experiment to probe the heterogeneous nuclear environment

**DOI:** 10.5194/mr-6-93-2025

**Published:** 2025-03-10

**Authors:** Sergei Kuzin, Victoriya N. Syryamina, Mian Qi, Moritz Fischer, Miriam Hülsmann, Adelheid Godt, Gunnar Jeschke, Maxim Yulikov

**Affiliations:** 1 Department of Chemistry and Applied Biosciences, ETH Zurich, Vladimir-Prelog-Weg 2, 8093 Zurich, Switzerland; 2 Voevodsky Institute of Chemical Kinetics and Combustion, Institutskaya str. 3, Novosibirsk 630090, Russia; 3 Faculty of Chemistry and Center for Molecular Materials (CM2), Bielefeld University, Universitätsstraße 25, 33615 Bielefeld, Germany

## Abstract

The intermolecular hyperfine relaxation-induced dipolar modulation enhancement experiment (ih-RIDME) is a pulse electron paramagnetic resonance (EPR) experiment that can be used to probe the properties of a nuclear spin bath in the vicinity of an unpaired electron. The underlying mechanism is the hyperfine spectral diffusion of the electron spin during the mixing block. A quantitative description of the diffusion kinetics being applied to establish the ih-RIDME data model allows one to extend this method to systems with heterogeneous nuclear arrangements assuming a distribution of the local nuclear densities. The heterogeneity can stem from the solvent or the intrinsic nuclei of a structurally flexible (macro)molecule. Therefore, the fitted distribution function can further serve as a method for heterogeneity characterization, quantification and structure-based analysis. Here, we present a detailed introduction to the principles of ih-RIDME application to heterogeneous systems. We discuss the spectral resolution, determination of the spectral diffusion parameters and influence of noise in the experimental data. We further demonstrate the application of the ih-RIDME method to a model spin-labelled macromolecule with unstructured domains. The fitted distribution of local proton densities was reproduced with the help of a conformational ensemble generated using the Monte Carlo approach. Finally, we discuss several pulse sequences exploiting the HYperfine Spectral Diffusion Echo MOdulatioN (HYSDEMON) effect with an improved signal-to-noise ratio.

## 1 Introduction

Among pulse electron paramagnetic resonance (EPR) experiments there are two large families of techniques that can be summarized under the names “hyperfine spectroscopy” and “electron–electron dipolar spectroscopy”. Pulse electron–electron dipolar spectroscopy (PDS) techniques have a longer upper distance limit and can even probe electron spin pairs separated by more than 10 nm [Bibr bib1.bibx11]. Thus, PDS techniques are able to provide long-range structural constraints. On the contrary, it is common to view hyperfine spectroscopy techniques in pulse EPR as “local structure” determination techniques, mainly targeting magnetic nuclei with resolved hyperfine splitting, within the shell of no more than 1 nm [Bibr bib1.bibx8]. The hyperfine techniques can further address nuclei beyond the 1 nm distance limit provided that their homonuclear interaction is negligible [Bibr bib1.bibx33].

In the case of many magnetic nuclei present in the vicinity of a paramagnetic centre, those that are weakly coupled cannot be well resolved in the nuclear magnetic resonance (NMR) spectrum, forming a so-called matrix peak positioned at the corresponding nuclear Zeeman frequency. The intensity of the matrix peak can be used as a measure of the total number of weakly coupled nuclei, e.g. in water accessibility experiments [Bibr bib1.bibx44].

In many samples, especially those related to biological systems or organic materials, there is a large number of magnetic nuclei of the same sort, typically protons, coupled to each other by dipolar and J -coupling. Such a network of coupled protons is called a proton spin bath, and it undergoes continuous quasi-stochastic local fluctuations in the spin states of individual protons. This process, called proton spin diffusion [Bibr bib1.bibx2], covers regions rather close to the paramagnetic centres present in the sample. As a result of proton spin diffusion, the electron spin senses a fluctuating hyperfine field formed by nearby protons and its resonance frequency also fluctuates. This process is called electron spectral diffusion [Bibr bib1.bibx25]. At short electron–proton distances, the proton spin diffusion is slowed down by the gradient of the electron's magnetic field and can be considered inactive on a timescale of a single shot in a pulse EPR experiment. The currently available evaluations suggest that the electron spectral diffusion process is sensitive to the protons in a range that may start between 0.5 and 1 nm and may end between 1.5 and 3 nm, depending on the typical strengths of proton–proton couplings which, in turn, are dependent on concentration [Bibr bib1.bibx28]. All of the protons within the blocked range only contribute to the full hyperfine spectrum of a given electron spin as a shift but do not participate in the electron spectral diffusion process.

The proton spin diffusion/electron spectral diffusion process, often being the main contribution to the phase memory time of organic paramagnetic centres in solid state [Bibr bib1.bibx42], can be detected in a stimulated electron spin echo experiment and its variations [Bibr bib1.bibx18]. The stimulated electron spin echo experiments exist in the form of a three-pulse electron spin echo envelope modulation (ESEEM) technique and modifications thereof as well as a relaxation-induced dipolar modulation enhancement (RIDME; Fig. [Fig Ch1.F1]a) technique and its variants. In the former class, the length of the longitudinal block is incremented, whereas the transverse part is usually kept constant. In the RIDME-based class, the increment relation is the opposite, which allows one to avoid the contribution of the electron spin longitudinal relaxation to the primary data. Thus, we focus on the determination of the parameters of the electron spectral diffusion/nuclear spin diffusion via the RIDME pulse sequence, and we refer to this experiment in this context as an intermolecular hyperfine (ih-) RIDME [Bibr bib1.bibx28].

The ih-RIDME traces, previously discussed in the literature as a RIDME background [Bibr bib1.bibx4], feature shape sensitivity to the local proton density and the mixing time (Fig. [Fig Ch1.F1]b). This allows the RIDME experiment to be exploited to probe the proton environment of the paramagnetic site. The dependence on the mixing time is related to the average strength of the nuclear–nuclear interaction and, hence, reflects the properties of the homonuclear coupling network. As a step further, the developed quantitative model for ih-RIDME enables the characterization of heterogeneous systems in terms of the distribution function of local proton densities. Thus, the ih-RIDME technique offers intermediate-range structural information, beyond the arrangement of ligands and solvent molecules in the coordination shell and/or in the first two to three solvation shells (which is typical information from other hyperfine spectroscopy techniques). This feature makes ih-RIDME the first hyperfine-based structural method for long-range structure determination (although the sensitive distance range for ih-RIDME is still substantially shorter than for PDS techniques). While global data fitting in ih-RIDME significantly improves the fit stability, it also raises a question about optimizing the signal quality and measurement time.

This article is organized as follows: after introducing the theory for ih-RIDME signals from heterogeneous proton distributions, we give an account of the spectral resolution. Next, we discuss the influence of the noise on the fit of ih-RIDME data, followed by a detailed discussion of the procedure for determining the fit parameters of the ih-RIDME kernel in the heterogeneous distribution case. After this, we turn to analyse the experimental ih-RIDME data obtained for a model compound containing one nitroxide unit with a highly anisotropic distribution of protons. Finally, before turning to conclusions, we give an account of selecting different versions of the RIDME experiment for better performance of ih-RIDME measurements.

**Figure 1 Ch1.F1:**
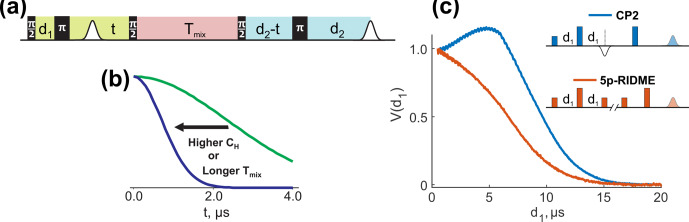
**(a)** The ih-RIDME pulse sequence. The pulse labels indicate their flip angles. Highlighted blocks correspond to the preparation (lime), diffusion (light red) and detection (light blue) functional elements of the sequence. **(b)** The ih-RIDME traces become steeper if the local nuclear concentration ( $C_{H}$ ) or the length of the diffusion block ( $T_{\text{mix}}$ ) increase. **(c)** Comparison of the echo intensity in the Carr–Purcell sequence (CP2) and the 5p-RIDME sequence as a function of the first interpulse delay ( $d_{1}$ ) measured in a protonated solution. The echo in CP2 has a maximum due to the dynamical decoupling effect in the presence of proton–proton coupling. In the case of RIDME, the echo monotonically decreases due to the presence of the longitudinal block. The details of the CP2 and 5p-RIDME measurements are presented in Sect. 3.

## 2 Theoretical background

The mixing block in the ih-RIDME experiment is an essential functional element. This can be demonstrated in a comparison of echo evolution in the Carr–Purcell 2 sequence (CP2), which is also the observer sequence in the four-pulse and five-pulse double electron–electron resonance (DEER) experiment and in the five-pulse RIDME experiment when the length of the first interpulse delay is incremented (Fig. [Fig Ch1.F1]c). Note that such an increment is unconventional for the RIDME experiment. In the CP2 experiment, the echo intensity is of non-monotonic behaviour with a distinct maximum (blue line in Fig. [Fig Ch1.F1]c). This effect is attributed to the presence of protons [Bibr bib1.bibx5]. During the CP2 experiment, electron magnetization continuously evolves in the transverse plane. The RIDME sequence can be seen as a CP2 sequence with an inserted $\pi /2-T_{\text{mix}}-\pi /2$ block, called the mixing block, during which the electron magnetization is aligned in the z direction. In the example in Fig. [Fig Ch1.F1]c, the mixing block is inserted at the position of the primary echo. As a result of such modification, the echo intensity monotonically decreases as a function of $d_{1}$ (orange line). This demonstrates that the mixing block has a strong influence on the echo intensity. Moreover, the observed intensity decay is indicative of the dissipative properties of spin dynamics during the mixing block, and this justifies the term “spectral diffusion” introduced in this section.

### 2.1 Longitudinal spectral diffusion

The ih-RIDME signal is a decay of the echo intensity in the RIDME experiment when the position of the mixing block shifts in the pulse sequence. The decay occurs due to the longitudinal spectral diffusion. This effect arises when the spectroscopic properties of the electron spin before and after the mixing block are not strictly correlated, leading to incomplete refocusing of spin–spin interactions. We neglect the contribution of the electron background [Bibr bib1.bibx24]; therefore, the interaction of interest in ih-RIDME is the hyperfine interaction with close nuclei. The Hamiltonian of this interaction in the secular approximation is as follows:

1 $${\hat{H}}_{\text{hf}}=\sum_{j=1}^{N}A_{j}{\hat{S}}_{z}{\hat{I}}_{z,j},$$

where S and I represent electron and nuclear spins, respectively; $A_{j}$ represents the hyperfine coupling constants; and N is the number of nuclei. The main example of nuclei in this work is protons, as they are the most abundant nuclei in applications such as biological studies and dynamic nuclear polarization (DNP) and have the largest gyromagnetic ratio; thus, their spin–spin interaction is the strongest. For protons at cryogenic temperatures, the driving force for the nuclear state evolution is the homonuclear dipolar coupling:

2 $${\hat{H}}_{\text{dd}}=\sum_{j\neq k}\omega_{j,k}\left(\frac{1}{2}{\hat{I}}_{z,j}{\hat{I}}_{z,k}-\frac{1}{4}{\hat{I}}_{j}^{+}{\hat{I}}_{k}^{-}\right),$$

where $\omega_{j,k}$ represents the nuclear dipolar coupling constants. The nuclear Zeeman Hamiltonian ${\hat{H}}_{\text{NZ}}=\sum_{j=1}^{N}\omega_{\text{H}}{\hat{I}}_{z,j}$ commutes with both ${\hat{H}}_{\text{hf}}$ and ${\hat{H}}_{\text{dd}}$ and is, therefore, not considered in the following discussion [Bibr bib1.bibx30].

The ih-RIDME pulse sequence (Fig. [Fig Ch1.F1]a) has three functional elements: preparation (highlighted in lime), mixing (light red) and detection (light blue) blocks. We assume, for generality, that the spin Hamiltonians during these blocks are ${\hat{H}}^{\text{(prep)}}$ , ${\hat{H}}^{\text{(diff)}}$ and ${\hat{H}}^{\text{(det)}}$ , respectively. By denoting the corresponding free evolution propagators as $U_{\text{prep}}$ , $U_{\text{diff}}$ and $U_{\text{det}}$ , the full expression for the ih-RIDME signal after propagation of the electron spin coherence ${\hat{S}}_{x}$ reads as follows:

3 $$V(t)\propto \langle {\hat{S}}_{x}\rangle =\mathrm{tr}({\hat{S}}_{x}{\hat{\sigma }}_{3}).$$

Here,

4σ^3=Udet(d2)UπUdet(d2-t)σ^2Udet(d2-t)†Uπ†Udet(d2)†;5σ^2=Uπ/2Udiff(Tmix)Uπ/2σ^1Uπ/2†Udiff(Tmix)†Uπ/2†;6σ^1=Uprep(d1+t)UπUprep(d1)S^xUprep(d1)†Uπ†Uprep(d1+t)†;

and $U_{\pi /2}$ and $U_{\pi }$ are the propagators of π/2 and π pulses, respectively.

Analytical evaluation of Eq. ([Disp-formula Ch1.E3]) is not feasible for a large number of nuclei and the full Hamiltonian (i.e. when ${\hat{H}}^{\text{(prep)}}={\hat{H}}^{\text{(diff)}}={\hat{H}}^{\text{(det)}}={\hat{H}}_{\text{hf}}+{\hat{H}}_{\text{dd}}$ ). In a simplified theoretical picture, we consider the hyperfine interaction and neglect the nuclear–nuclear interaction during the preparation and detection part of the RIDME pulse sequence:

7 $${\hat{H}}^{\text{(prep)}}={\hat{H}}^{\text{(det)}}={\hat{H}}_{\text{hf}}\;.$$

Such an approximation means that the magnetic state of the nuclear bath $|M\rangle ={⊗}_{j=1}^{N}|m_{I,j}\rangle$ , constructed from Zeeman states of individual nuclei, is stationary during the preparation and detection blocks. Consequently, it allows us to characterize the interaction of the electron spin with the nuclear reservoir in state |M〉 using the so-called hyperfine field (in angular frequency units):

8 $$\omega_{\text{hf}}=\langle M|\sum_{j=1}^{N}A_{j}{\hat{I}}_{z,j}|M\rangle =\sum_{j=1}^{N}A_{j}m_{I,j},$$

which is conserved during the electron spin transverse evolution [Bibr bib1.bibx28]. The hyperfine field is the resonance offset of the electron spin due to interaction with all of the nuclei. This value depends on both nuclear coordinates via $A_{j}$ and on the magnetic state of the nuclei via $m_{I,j}$ and is thereby distributed. The distribution of the hyperfine fields, obtained by sampling all nuclear bath states |M〉 , is called a hyperfine spectrum $\rho (\omega_{\text{hf}})$ . This function describes the distribution of resonance offsets in an ensemble of electron spins with fixed nuclear coordinates but different nuclear spin states. The hyperfine spectrum also depends on the nuclear arrangement around the electron spin. Its mean value is not detectable in the pulse experiment because such a constant offset is refocused at the echo detection. In the statistical limit, we approximate the shape of the hyperfine spectrum as Gaussian:

9 $$\rho \left(\omega_{\text{hf}}\right)\approx \frac{1}{\sqrt{2\pi }\sigma }\mathrm{exp}\left(-\frac{{\left(\omega_{\text{hf}}-\bar{\omega }\right)}^{2}}{2\sigma^{2}}\right)\;.$$

Given that the offset $\bar{\omega }$ is not detectable, the standard deviation σ is sufficient to represent the hyperfine spectrum.

We can relate σ to some geometric properties of a nuclear reservoir. Consider N nuclei with coordinates $C=\{r_{j}{\}}_{j=1}^{N}$ and with the same nuclear spin I . Assuming that the spectral diffusion is ergodic, i.e. that all nuclear spin states are statistically equivalent, the variance of the hyperfine spectrum is the second central moment of $\omega_{\text{hf}}$ . For computation, we plug the definition into Eq. ([Disp-formula Ch1.E8]) and assume that the nuclear spin quantum numbers are uncorrelated. Therefore, the variance of the sum is the sum of terms' variances, and we obtain the following:

10 $$\sigma^{2}(C)=\sum_{j=1}^{N}\frac{1}{2I+1}\sum_{m_{I}=-I}^{I}A_{j}^{2}m_{I}^{2}=\frac{I(I+1)}{3}\sum_{j=1}^{N}A_{j}^{2}\;.$$

We can transform this expression in the case of uniformly distributed nuclei in space at distances from r=R to r=+∞ and apply the point-dipole approximation for the hyperfine coupling:

11 $$\begin{array}{rl} \sigma^{2}(R,\infty ) & =\frac{I(I+1)}{3}{\left(\frac{\hbar \mu_{0}\gamma_{e}\gamma_{n}}{4\pi }\right)}^{2}\int_{R}^{+\infty }dr\;4\pi r^{2}C_{H}\\ & \times \int_{0}^{\pi }\frac{{\left(1-3{\mathrm{cos}}^{2}\theta \right)}^{2}}{r^{6}}\frac{\mathrm{sin}\theta }{2}d\theta =\frac{4\pi }{3}B^{2}\frac{C_{H}}{R^{3}}, \end{array}$$

where the constant $B=|\hbar \mu_{0}\gamma_{e}\gamma_{n}/4\pi |\cdot (4I(I+1)/15{)}^{1/2}$ is 0.222 MHz nm^3^ for protons and $C_{H}$ ( ${\mathrm{nm}}^{-3}$ ) is the proton number density. The derivation of this equation and the derivation of $\sigma^{2}$ for different topologies of proton distributions are presented in Appendix [App App1.Ch1.S1].

We restrict further considerations to systems and measurement conditions in which nuclear longitudinal ( $T_{1n}$ ) and zero-quantum relaxation ( $T_{2,ZQ}$ ) can be neglected on the timescale of the RIDME experiment. Under these assumptions, not all protons around the electron spin contribute to the spectral diffusion processes. The internuclear coupling of close protons is not strong enough to mix the hyperfine levels and induce the flip-flop transition. If the difference in the hyperfine couplings in a pair of protons is much greater in absolute value than the nuclear–nuclear interaction, we refer to such protons as being strongly coupled to the electron. In terms of the nuclear-pair ESEEM model, the ESEEM modulation depth is close to zero [Bibr bib1.bibx18]. In the multi-nuclear spin system, there is no known general analytical relation between the parameters of the hyperfine and nuclear Hamiltonians and the probability of nuclear transitions. We suggested using the value of R that fits the experimentally determined values of σ , as in Eq. ([Disp-formula Ch1.E11]). We call such a characteristic value a spin diffusion radius, $R_{\text{sd}}$ . It represents the region of electron–nuclear distances where protons have the strongest contribution to spectral diffusion via the mechanism of spin diffusion.

Upon proton dilution, achieved using deuterated water and glycerol, the following was found [Bibr bib1.bibx28]:

12 $$\sigma \propto C_{H}\;.$$

The proportionality factor $\sigma /C_{H}$ for the water–glycerol mixture ( 1:1 v/v ) is 0.0215 MHz L mol^−1^ [Bibr bib1.bibx28] if the proton concentration is used or 0.0357 MHz nm^3^ if the number density is used. Equation ([Disp-formula Ch1.E11]) shows $\sigma \propto \sqrt{C_{H}}$ ; consequently, the spin diffusion radius depends on proton concentration as $R_{\text{sd}}\propto C_{H}^{-1/3}$ . The value of the radius depends on the typical distance between the neighbouring protons, as this distance affects the nuclear–nuclear coupling constants in ${\hat{H}}_{\mathrm{dd}}$ . The weaker the average nuclear dipolar interaction, the larger the spin diffusion radius. For a fully protonated water–glycerol mixture, we experimentally determined $R_{\text{sd}}=1.36\;\mathrm{nm}$ [Bibr bib1.bibx28].

To simplify the description of the spin dynamics during the mixing block, as the ${\hat{H}}_{\text{dd}}$ cannot be omitted now, we use a diffusion-like approximation based on a formalism of the magnetization spectrum [Bibr bib1.bibx28]. The latter is an ensemble function that specifies the phase evolution of electron spin packets with a certain hyperfine field accounting for their statistical weights. The magnetization spectrum also depends on the length of the preparation and diffusion blocks ( t and T , respectively), which is reflected in the notation $\mu_{t}(\omega_{\text{hf}},T)$ . Given the approximation in Eq. ([Disp-formula Ch1.E7]), the spin packet with the hyperfine field $\omega_{\text{hf}}$ , after the preparation time t , starts the mixing block with the phase $\omega_{\text{hf}}t$ which is reflected as follows:

13 $$\mu_{t}(\omega_{\text{hf}},0)=e^{i\omega_{\text{hf}}t}\rho (\omega_{\text{hf}})\;.$$

The evolution of this function during the mixing block is approximated using the following differential equation:

14 $$\begin{array}{rl} \frac{\partial \mu \left(\omega_{\text{hf}},T\right)}{\partial T} & =D(\rho \left(\omega_{\text{hf}}\right)\mu_{\omega \omega }^{\prime \prime }\left(\omega_{\text{hf}},T\right)\\ & -\rho^{\prime \prime }\left(\omega_{\text{hf}}\right)\mu \left(\omega_{\text{hf}},T\right)), \end{array}$$

where D ( ${\mathrm{freq}}^{3}/\mathrm{time}$ ) is the spectral diffusion coefficient. After the mixing block, the detection block follows, and the ih-RIDME signal is expressed via the magnetization spectrum as follows:

15 Rt;Tmix=Re∫-∞+∞μtωhf,Tmixe-iωhftdωhf.

Here, we use the symbol R(t) for the echo intensity, instead of V(t) as in Eq. ([Disp-formula Ch1.E3]), to emphasize that a simplified model Hamiltonian is used for the transverse evolution.

The solution $R(t;T_{\text{mix}})$ can be represented in several mathematical forms (see Supplement S2 and [Bibr bib1.bibx28]). We summarize two main properties. First, the solution is a family of master decays as functions of the product σt , and this family of master decays is parameterized by mixing time $T_{\text{mix}}$ . This means that the shapes of the ih-RIDME decays with different σ are congruent. The decays at a given width σ are directly recalculated from the master functions by stretching the argument. Second, the spectral diffusion kinetics is naturally parameterized by $\kappa =D/\sigma^{3}$ ( ${\mathrm{time}}^{-1}$ ). So far, there is no ab initio derivation of this parameter from the spin Hamiltonian. Nevertheless, it can be interpreted qualitatively. It was found previously [Bibr bib1.bibx28] that κ is invariant upon isotope dilution of homogeneous solutions. In this case, the density of the nuclear–nuclear coupling network per single nucleus also remains constant. Such density can be interpreted as a measure of the average number of strong homonuclear contacts per nucleus. Should this number be reduced, the kinetics of spectral diffusion slows down and the ratio $D/\sigma^{3}$ correspondingly decreases. As a model example, a $-({\mathrm{CH}}_{2}{)}_{n}-$ fragment in a deuterated solution can be considered. The homonuclear coupling network approximately follows the 1D geometry of the chain and a reduction in the spectral diffusion kinetics is thus expected.

### 2.2 ih-RIDME data model

The ih-RIDME signal in the 5p-RIDME experiment at sufficiently long mixing times has the following product structure: [Bibr bib1.bibx28]

16 $$V\left(t;T_{\text{mix}},d_{1},d_{2}\right)\approx R\left(t;T_{\text{mix}}\right)\cdot F(t;d_{1},d_{2}),$$

where $d_{1}$ and $d_{2}$ are the static delays of the pulse sequence. The factor R(t) is called a longitudinal factor. It depends only on the position of the mixing block and the mixing time, not on the pulse sequence's static auxiliary delays. This factor was explained in the longitudinal spectral diffusion model described in Sect. 2.1. The second factor F(t) is independent of the mixing time and depends on $d_{1}$ and $d_{2}$ . To give it a quantitative explanation, we need to include the nuclear dipolar interaction in the Hamiltonian during the transverse part of the RIDME sequence:

17 $${\hat{H}}^{\text{(prep)}}={\hat{H}}^{\text{(det)}}={\hat{H}}_{\text{hf}}+{\hat{H}}_{\text{dd}}\;.$$

The evolution of the electron spin coherence with this Hamiltonian cannot be solved analytically. By using the perturbation analysis [Bibr bib1.bibx30], we established some analytical properties of the solution. It was shown that the ih-RIDME factorization, as in Eq. ([Disp-formula Ch1.E16]), holds if the mixing times exceed the full decay of the Hahn echo. If this is not satisfied, the shape of F(t) becomes dependent on the mixing time. Throughout this work, we refer to F(t) in its limiting form for $T_{\text{mix}}\gg T_{m}$ . The shape of F(t) has the same congruence property for different values of σ , as R(t) ; i.e. F also depends on the product σt [Bibr bib1.bibx28].

In heterogeneous systems, every electron spin may be in a different nuclear environment and, hence, have a different hyperfine spectrum. Therefore, the width of the hyperfine spectrum becomes a distributed value described by a distribution function p(σ) . The ih-RIDME representation is generalized in this case as follows:

18 $$V\left(t;T_{\text{mix}},d_{1},d_{2}\right)=\int_{\sigma_{\text{min}}}^{\sigma_{\text{max}}}p(\sigma )V_{\sigma }\left(t;T_{\text{mix}},d_{1},d_{2}\right)d\sigma ,$$

where $V_{\sigma }$ is an ih-RIDME trace corresponding to a hyperfine spectrum with a standard deviation of σ [Bibr bib1.bibx29].

### 2.3 Spectral diffusion beyond protons

The description of spectral diffusion in ih-RIDME driven by the homonuclear coupling potentially applies to any nuclei with a non-zero magnetic moment $\mu_{\text{nuc}}$ . The relevant candidates are, for example, ${}^{19}F$ ( $\mu_{\text{nuc}}({}^{1}H)/\mu_{\text{nuc}}({}^{19}F)=1.06$ ), ${}^{13}C$ ( $\mu_{\text{nuc}}({}^{1}H)/\mu_{\text{nuc}}({}^{13}C)=3.98$ ) and ${}^{31}P$ ( $\mu_{\text{nuc}}({}^{1}H)/\mu_{\text{nuc}}({}^{31}P)=2.47$ ). These are examples of nuclei whose relaxation can be neglected at cryogenic temperatures on the timescale of a RIDME experiment. The nuclei with a lower gyromagnetic ratio feature slower spectral diffusion kinetics. Indeed, the hyperfine coupling scales as the first power $\mu_{\text{nuc}}$ , whereas the homonuclear dipolar coupling scales as a square ( $\mu_{\text{nuc}}^{2}$ ). Assuming a uniform distribution of nuclei around the electron spin, we obtain the generalization of Eq. ([Disp-formula Ch1.E12]) (the derivation is presented in Appendix [App App1.Ch1.S2]):

19 $$\sigma \propto |\mu_{\text{nuc}}{|}^{11/8}C_{n},$$

where $C_{n}$ is the nuclear concentration or density. As the ih-RIDME kernel depends on the product σt , the reduction in σ means that longer traces are necessary to achieve the same decay degree. As, for low- γ nuclei, the nuclear dipolar interaction decreases faster than the hyperfine interaction, the diffusion parameter $D/\sigma^{3}$ is expected to decrease, although the exact dependence is not known. Reduction in $D/\sigma^{3}$ means that the spectral diffusion effect in ih-RIDME builds up more slowly with mixing time.

In a mixture of different magnetic nuclei, upon neglecting the heteronuclear dipolar interaction, the ih-RIDME trace is approximated by a product of signals corresponding to each type of magnetic nuclei:

20 $$V\left(t;T_{\text{mix}}\right)\approx \prod_{j}V_{j}\left(t;T_{\text{mix}}\right)\;.$$

Consequently, to better observe the spectral diffusion induced by the low- γ nuclei, one needs to reduce or eliminate the competitive dynamics from the higher- γ nuclei, e.g. by means of isotope replacement or chemical substitution.

## 3 Experimental

The synthesis and characterization of model compound 1 is presented in the Supplement. For our tests, this model compound was dissolved in $H_{2}O$ + $D_{2}O$ + $D_{8}$ -glycerol (40 % glycerol v/v ) to the concentration of 25 µ M, and four samples with bulk solvent proton concentrations of 0, 11, 22 and 42 M (Table [Table Ch1.T1]) were prepared. The pulse EPR measurements were performed at Q band ( $\nu_{\mathrm{mw}}=34.5\;\mathrm{GHz}$ ) at 50 K. The series of RIDME traces were recorded at the maximum field position with $t_{\pi /2}=12\;\mathrm{ns}$ and $t_{\pi }=24\;\mathrm{ns}$ , with $d_{2}=4.2$ µ s, and for $T_{\mathrm{mix}}$ from 15 to 480 µ s. For the reference division, traces with $T_{\mathrm{mix}}=30$ µ s (samples 1 and 2) and $T_{\mathrm{mix}}=15$ µ s (samples 3 and 4) were used.

**Table 1 Ch1.T1:** Composition of solutions of model compound 1 in a water–glycerol mixture (60 : 40 v/v ) to study the solvent contrast.

Symbol	$C_{H}\text{(solvent)}$ (M)	$T_{\text{mix}}^{\text{ref}}$ ( µ s)
1	0	30
2	11.4	30
3	22.1	15
4	41.7	15

The pulse EPR measurements in Figs. [Fig Ch1.F1]c and [Fig Ch1.F10] were done with a solution of 50µ M TEMPO (2,2,6,6-tetramethylpiperidin-1-yl-oxyl) at Q band at 50 K. The solvent is a mixture of $H_{2}O$ and $D_{8}$ -glycerol ( 60:40 v/v ) with a total proton concentration of $C_{\text{H}}=56$ M. The CP2 trace was recorded as $\pi /2-d_{1}-\pi -(d_{1}+d_{2})-\pi -d_{2}-\text{echo}$ with a fixed $d_{2}=4.2$ µ s and variable $d_{1}$ . The RIDME echo vs. delay $d_{1}$ was recorded with parameters $d_{2}=4.2$ µ s and $T_{\text{mix}}=30$ µ s.

## 4 Discussion

### 4.1 Origins of the nuclear heterogeneity

We can indicate several origins of nuclear heterogeneity. The simplest example is fractional heterogeneity; i.e. when a spin probe is distributed between phases or microphases, each of which is characterized by a homogeneous proton distribution. This may be a mixture of two solutions with a phase boundary, a biphasic solution of a (bio)molecule prone to condensate [Bibr bib1.bibx10] or paramagnetic sites in a material with different solvent accessibility. In these examples, at each location, the proton distribution is homogeneous and the local concentration is thus well-defined but its value is distributed (different at different locations).

A different type of heterogeneity is local (or structural) inhomogeneity. This case can be associated with the conformational flexibility and condensation interaction of spin-labelled macromolecules or a labelled macromolecule in a deuterated buffer. Upon conformational flexibility, the distribution of the proton environment arises due to the different variants of macromolecule shape, e.g. backbone conformation of a spin-labelled intrinsically disordered protein (IDP). This situation is typical when macromolecules weakly interact forming aggregates or condensates of variable density and homogeneity [Bibr bib1.bibx29]. The proton distribution in such systems can be substantially irregular and a homogeneous proton concentration is thus undefined. Therefore, we can generally use a width of the hyperfine spectrum $\rho (\omega_{\text{hf}})$ to quantify a proton configuration with the help of Eq. ([Disp-formula Ch1.E10]). The distribution of nuclear configurations, which can manifest in different numbers of nuclei N and their coordinates, reflects in a distribution of σ . This principle is illustrated in Fig. [Fig Ch1.F2]. As we assume to work in a solid state, the nuclear coordinates do not change. Therefore, the mean value of the hyperfine field for each configuration C is not observable ( $\bar{\omega }$ in Eq. [Disp-formula Ch1.E9]), and we will work with a distribution of standard deviations, p(σ) .

**Figure 2 Ch1.F2:**
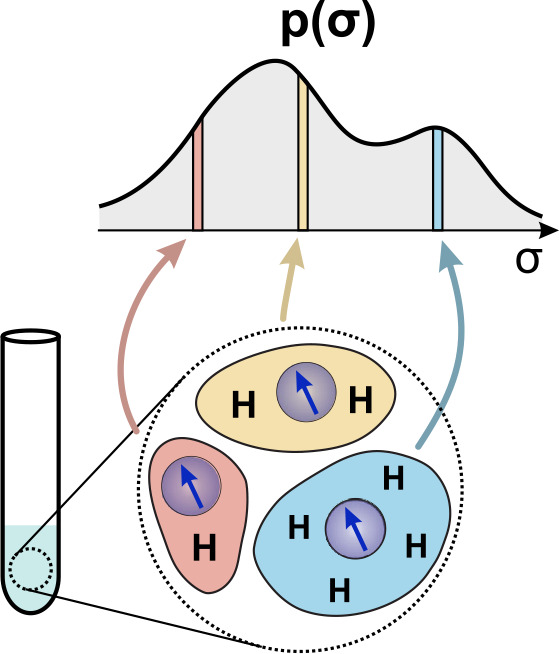
Schematic representation of a system with proton heterogeneity and its representation as a distribution of σ values related to the local proton densities (see the main text for details).

An anisotropic proton distribution, as is typical of structural inhomogeneity, obtains a non-zero width in the σ scale when averaged over all orientations. We demonstrate this in a simulation. To this end, we generated a spherical proton cloud of radius R , local concentration $C_{H}$ and distance from an electron spin d , as depicted in Fig. [Fig Ch1.F3]. Angle θ parameterizes the position of the cloud in spherical coordinates. Specification of the polar angle φ is not required due to the cylindrical symmetry of the problem. This model pictures an anisotropic spatial distribution of nuclei. The distribution of geometric parameters, such as electron–nuclear dipolar angles, is not uniform on a corresponding unit sphere. Consequently, the hyperfine spectrum of the individual configuration is expected to be narrower than that of the purely isotropic one, and its shape varies when the configuration is rotated in space. If we expect a Gaussian shape of $\rho (\omega_{\text{hf}})$ in each orientation, then we consider only the variation in its standard deviation.

For the chosen values of d (6, 4, 3 and 2 nm), we sampled angle θ over the interval [0;π] and computed the standard deviations of the hyperfine spectra with Eq. ([Disp-formula Ch1.E10]). We used a spherical cloud of R=1.5 nm and $C_{H}=20$ M in the computation, which resulted in 170 protons. The hyperfine couplings were calculated using the point-dipole approximation. The resulting distributions p(σ) are shown in Fig. [Fig Ch1.F3]. We can see that the distributions resemble half of the Pake pattern for d=6 nm and 4 nm. This means that the size of the proton cloud is negligible compared to the distance d and that the cloud can be approximately represented by a point magnetic moment. Another indication is that the distribution p(σ) attains values close to zero. This corresponds to the angles θ close to the magic angle $\Theta_{M}=\mathrm{arccos}(1/\sqrt{3})$ , and the scattering of electron–nuclear dipolar angles is again negligible.

The situation changes when the proton cloud is close to the electron and the point approximation for the proton cloud is no longer applicable. This is exemplified in Fig. [Fig Ch1.F3] for d=3 and d=2nm where the shape of the distributions deviates from the Pake pattern.

**Figure 3 Ch1.F3:**
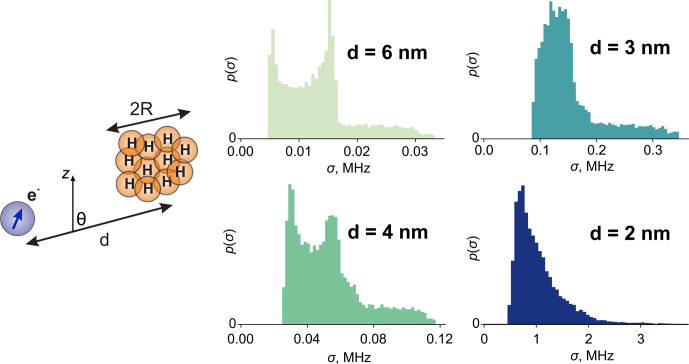
A model of a proton cloud of radius R with its centre at a distance d from the electron. The histograms are the distributions of the hyperfine spectrum standard deviation ( σ ) for different indicated values of d . For the simulation, the values R=1.5nm and $C_{H}=20$ M were used.

The main conclusion from this computational exercise is that the distributions of proton densities include several sources of broadening that are not possible to a priori disentangle. The structural problem in ih-RIDME is more complex than a two-spin interaction in PDS. The PDS signal in the frequency domain is a superposition of Pake patterns with different splitting parameters. Such a spectrum, if the underlying distance distribution is sufficiently broad, may look like a smooth, feature-less function. Further, a distance between two spins is a clear geometric invariant upon global frame rotation. Consequently, upon neglecting the exchange interaction, the dipolar spectrum can be deconvoluted from the Pake pattern by various mathematical means [Bibr bib1.bibx20], and the result corresponds to a distance distribution. As the ih-RIDME method, by design, analyses interaction with many nuclear spins, the description of a geometric configuration by a single invariant is not feasible. This obstacle affects the use of ih-RIDME data in ensemble-based computation methods, as the intermediate processing result in the form of distribution function p(σ) may be less intuitive than the distance distribution in PDS. In the cases in which the nuclear distribution around the electron spin is close to isotropic, the value of σ can be related to the nuclear density. This is relevant for the first kind of heterogeneity, and the function p(σ) has the meaning of the distribution of local nuclear density. Strictly speaking, this meaning is not applicable to systems with strongly anisotropic nuclear arrangements with respect to the electron spin, like spin-labelled macromolecules. There, the distribution of conformers and the broadening from the orientational average of the anisotropic nuclear placement may have equal contributions to the experimentally determined distributions p(σ) .

### 4.2 Simplified ih-RIDME kernel

The representation of the ih-RIDME kernel in the exact form is not always convenient (see Eq. [Disp-formula Ch1.E15] and Supplement S2). We found an approximation for it that allows us to discuss its analytical features. The approximation reads as follows:

21 $$R\left(t;T_{\text{mix}}\right)\approx \mathrm{exp}\left(-\alpha \left(T_{\text{mix}}\right)\sigma^{2}t^{2}\right),$$

with

22 $$\alpha \left(T_{\text{mix}}\right)\approx 1-\mathrm{exp}\left(-0.245(D/\sigma^{3})T_{\text{mix}}\right)\;.$$

$\alpha (T_{\text{mix}})$ is a growing function from 0 to 1 corresponding to the increasing steepness of the decay for longer mixing times. The quality of this approximation is shown in Supplement S2.

In [Bibr bib1.bibx28], we showed that the factor F(t) in 5p-RIDME can be approximated by a Gaussian decay:

23 $$F(t)\approx \mathrm{exp}\left(-\beta \sigma^{2}t^{2}\right)\;.$$

The shape of F is sensitive to the parameters of the RIDME sequences. In particular, parameter β has a weak dependence on delays $d_{1}$ and $d_{2}$ . So far, the full-Hamiltonian calculation of the transverse factor is not achieved; therefore, we will rely on the model functions approximating it.

By combining Eqs. ([Disp-formula Ch1.E21]) and ([Disp-formula Ch1.E23]), we obtain a simplified form for $V_{\sigma }(t)$ :

24 $$V_{\sigma }(t)\approx \mathrm{exp}\left(-\left(\alpha \left(T_{\text{mix}}\right)+\beta \right)\sigma^{2}t^{2}\right)\;.$$

This approximation implies that the ih-RIDME traces of homogeneous ensembles are close to Gaussian decays. The Gaussian shape is a direct consequence of Gaussian approximation for the hyperfine spectrum (as in Eq. [Disp-formula Ch1.E9]). The curvature of the decay ( $(\alpha (T_{\text{mix}})+\beta )\sigma^{2}$ ) monotonically increases with mixing time and levels off at high $T_{\text{mix}}$ . The rate of growth is parameterized by $D/\sigma^{3}$ . After the reference division, the parameter β is eliminated and the decay curvature is $(\alpha (T_{\text{mix}})-\alpha (T_{\text{mix}}^{\text{ref}}))\sigma^{2}$ .

In further discussion, we will use a general symbol $K(\sigma t;T_{\text{mix}})$ for the ih-RIDME kernel without specifying whether an approximate or exact form is taken.

### 4.3 Fitting

The dataset for fitting consists of several normalized ih-RIDME traces measured with different mixing times and a reference trace with the shortest mixing time. The time-domain traces are normalized to the zero time $V(t=0;T_{\text{mix}})$ . It is known that, at long mixing times comparable with electron $T_{1}$ , a zero-time echo-crossing artefact becomes visible [Bibr bib1.bibx23]. The intensity of this artefact increases with the mixing time (see Fig. S8 in Supplement S5) and the intensity normalization step must exclude it. The set of mixing times can be chosen as a geometric series with a common ratio of 2, i.e. $T_{\text{ref}},2T_{\text{ref}},4T_{\text{ref}},8T_{\text{ref}}$ etc. The common ratio of 2 allows for a simple implementation of a 2D-experiment acquisition using the PulsSPEL scripting language employed by commercial Bruker spectrometers. The traces are divided by the reference trace to remove RIDME artefacts that are not covered by the considered model (see [Bibr bib1.bibx34]; and discussion in Supplement S5) and fitted by a ratio of computed traces with a test distribution function p(σ) .

The optimization problem is mathematically formulated as follows:

25 $$\begin{array}{rl} p(\sigma ) & =\mathrm{arg}\underset{p⩾0}{min}\{\sum_{i=1}^{M-1}w_{i}\|\frac{V\left(t;T_{\text{mix},i}\right)}{V\left(t;T_{\text{mix},\text{ref}}\right)}\\ & -\frac{\int K\left(\sigma t;T_{\text{mix},i}\right)p(\sigma )d\sigma }{\int K\left(\sigma t;T_{\text{mix},\text{ref}}\right)p(\sigma )d\sigma }\|\}, \end{array}$$

where M is the number of recorded traces and $w_{i}⩾0$ represents weights. In all further examples, the optimization weights are set to 1 . The integration is meant from $\sigma_{\text{min}}$ to $\sigma_{\text{max}}$ . Furthermore, the parameters $D/\sigma^{3}$ and β may be additionally optimized. In principle, it is feasible to assume that heterogeneous systems with a broad or multicomponent distribution p(σ) may be characterized by a discreet or continuous distribution of these parameters. Here, we do not consider this case and assume single values for $D/\sigma^{3}$ and β .

The numeric tests below were performed with a home-written optimization algorithm to facilitate the investigation of the fitting history. The algorithm is written in Python and implements a gradient-descending least-squares fitting of a series of ih-RIDME traces.

**Figure 4 Ch1.F4:**
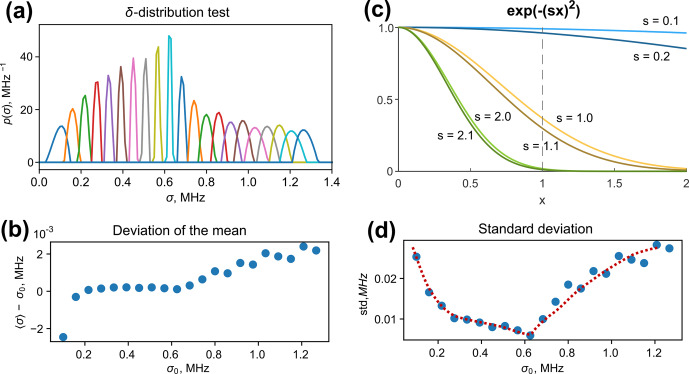
**(a)** Fitted distributions of the δ -condition test. **(b)** The deviation of the mean value of a fitted distribution from $\sigma_{0}$ for different tested $\sigma_{0}$ . **(d)** The standard deviations of the fitted distributions. The dashed line highlights the trend. **(c)** A plot of three pairs of Gaussian decays $\mathrm{exp}(-(s\cdot x{)}^{2})$ of variable x parameterized by s ; the higher the s value, the lower the shape sensitivity to the shift in s . This observation explains the fit broadening of the δ test at large values of $\sigma_{0}$ (see the text for details).

#### 4.3.1 The δ -initial condition

We tested the convergence of the fitted distribution in the case of homogeneous systems. Such systems would be described by a single proton concentration, i.e. δ -shape distribution p(σ) , as in Eq. ([Disp-formula Ch1.E26]). This test is motivated by noting that, in contrast to the two-spin dipolar kernel in PDS [Bibr bib1.bibx35], the ih-RIDME kernel (see Eq. [Disp-formula Ch1.E24]) is represented by smooth monotonic decays without characteristic minima or maxima. The traces corresponding to close points in the σ domain are also close in the time domain in the standard function metric. This is a known problem of the inverse Laplace transform. However, the ih-RIDME inversion deals with global Laplace inversion of multiple decays, the relative steepness of which is essentially controlled by a single parameter $\left(D/\sigma^{3}\right)$ . This condition stabilizes the ill-posed problem. However, as the problem does not become well-posed, an uncertainty in the peak width can be anticipated.

We generated a set of five ih-RIDME traces assuming a certain value of $\sigma =\sigma_{0}$ and fitted them using a model-free distribution model, as in Eq. ([Disp-formula Ch1.E18]). The time axis of RIDME traces was in 0 to 4 µ s. The mixing times were $T_{\text{mix}}=60$ , 120, 240 and 480 µ s, and $T_{\text{mix, ref}}=30$ µ s. The value of $\sigma_{0}$ was scanned in a range of 0.1–1.3 MHz.

26 $$p(\sigma )=\delta \left(\sigma -\sigma_{0}\right)$$

The resulting normalized fitted distributions are plotted in Fig. [Fig Ch1.F4]a. We observe that tests with low $\sigma_{0}$ and high $\sigma_{0}$ converged to broadened distributions. The mean values are in good agreement with the tested values of $\sigma_{0}$ , as can be seen from Fig. [Fig Ch1.F4]b. The relative deviation did not exceed 2 %. This indicates the high reliability of the distribution's mean value determination. The fitted standard deviation exhibits a minimum at $\sigma_{0}\approx 0.64$ MHz and increases at low and high values of $\sigma_{0}$ . We can explain this behaviour by analysing the ih-RIDME kernel in a Gaussian approximation. In Fig. [Fig Ch1.F4]c, we plot abstract Gaussian functions $\mathrm{exp}(-(s\cdot x{)}^{2})$ for s=0.1 (light blue), s=1.0 (light yellow) and s=2.0 (light green). Decays in darker hues correspond to functions where s is incremented by 0.1 . The parameter s in these examples corresponds to σ , albeit not precisely, as ih-RIDME traces are additionally affected by $\alpha (T_{\text{mix}})$ . For s=0.1 , a small increment of s has a weak effect at x<1 . Thus, if the trace length is insufficient (e.g. x<1.0 , as marked using a vertical dashed line to guide the reader's eye), the sensitivity of the trace shape to the change in s is weak. In the range of 1<x<2 , the trace difference is much larger. This example explains the impact of trace length on the resolution in a low- σ domain ( $\sigma_{0}<0.25$ MHz in Fig. [Fig Ch1.F4]a and d). Under the conditions of insufficient trace length, only the mean value of the distribution p(σ) can be precisely obtained but not the full shape. To increase the resolution in this region, one needs to record longer RIDME traces. The case s=1.0 in Fig. [Fig Ch1.F4]c shows the highest shape response to the variation in s and, hence, the highest resolution in the s domain (as an analogue the of σ domain). For s=2.0 and 2.1 , the trace difference is again small, and this is an intrinsic property because the extension of trace length does not improve the trace distinction due to their decay to zero. Such an observation is reflected in the fit broadening in the high- $\sigma_{0}$ part ( $\sigma_{0}>0.8$ MHz in Fig. [Fig Ch1.F4]a and d).

Overall, we observe a rather good reproducibility of the δ distributions in the data processing. Global fitting of ih-RIDME data stabilizes the inverse problem. The mean values of fitted distributions can be reliably determined, while width uncertainties may remain. In general, given the fixed length of the traces, there is a mid- σ region with the highest shape reliability. The size of this region can be extended into the low- σ regions by acquiring longer RIDME traces (i.e. optimizing $t_{\text{max}}$ ).

#### 4.3.2 White Gaussian noise test

We simulated ih-RIDME data assuming a Gaussian distribution p(σ) with the centre $\sigma_{0}=0.5$ MHz and standard deviation 0.18 MHz and added Gaussian white noise:

27 $$V_{\text{sim}}\left(t;T_{\text{mix}}\right)=V_{\text{RIDME}}\left(t;T_{\text{mix}}\right)+\Delta V(t).$$

Here,

28 $$V_{\text{RIDME}}\left(t;T_{\text{mix}}\right)=\int_{\sigma_{\text{min}}}^{\sigma_{\text{max}}}K\left(\sigma t;T_{\text{mix}}\right)p(\sigma )d\sigma ,$$

where $\sigma_{\text{min}}=0$ MHz, $\sigma_{\text{max}}=1$ MHz and ΔV(t) is white noise with a Gaussian distribution of the amplitudes. The noise function was centred ( $\bar{\Delta V(t)}=0$ ). The signal-to-noise ratio (SNR) of the simulated trace was defined as $V_{\text{RIDME}}(0;T_{\text{mix}})/\sqrt{\bar{(\Delta V{)}^{2}}}$ . The number of traces and the mixing times were the same as in the δ -distribution test. Figure [Fig Ch1.F5]a displays the fitting results with a progressing signal-to-noise ratio. The original distribution is given as a grey area, and lines of different colours correspond to the optimal fits with various noise levels. The noise-free fit almost exactly reproduces the target distribution, whereas the fitting of noisy data progressively deviates from it. The ih-RIDME kernel is rather smooth and should work as an effective low-pass filter in the time domain; however, in our tests starting from a SNR of 33, the fitted distributions develop some false shape features.

**Figure 5 Ch1.F5:**
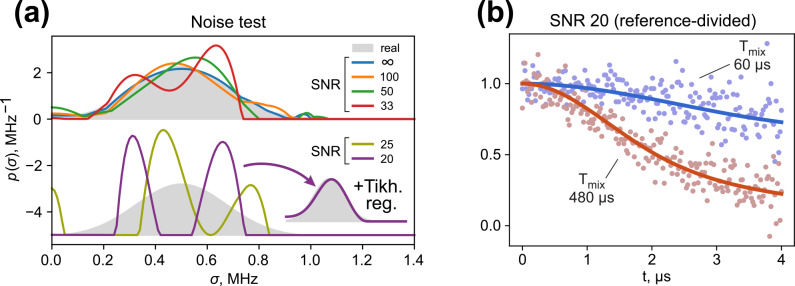
Influence of the noise level in the ih-RIDME traces on the fitting result tested on the generated datasets. **(a)** Deviation of the fit result from the distribution used to generate ih-RIDME traces (grey) at different SNRs. At SNRs of 25 and 20 the fitted distribution separates into two components. Tikhonov regularization (adding a penalty function of smoothness of the distribution's second derivative) mitigates the splitting. **(b)** Reference-divided traces (dots) and their fits (solid lines) in the case of a SNR of 20. The shown examples correspond to $T_{\text{mix}}=60$ µ s (slower decay) and $T_{\text{mix}}=480$ µ s (faster decay). After the trace division, the effective noise level is notably elevated and the fitted distribution may converge to a distorted shape.

Due to trace division, the noise level of the reference-divided traces approximately doubles (Fig. [Fig Ch1.F5]b). Consequently, the extreme cases of SNRs of 25 and 20 appear to yield a false multicomponent property of the fitted distribution function. We note that the mean value and the standard deviation of the distribution are stable upon adding noise to the data (see Fig. S4 in Supplement S3). Accurate determination of the distribution shape using a model-free approach in this example requires SNR values of 50 or higher. This can be efficiently helped by using derivative-based regularization techniques or others [Bibr bib1.bibx16]. We applied a penalty function – a norm of the distribution's second derivative - when fitting the traces with a SNR of 20 and were able to obtain a fit result close to the original distribution (inset in Fig. [Fig Ch1.F5]a). For samples with a weak EPR signal, one may opt to record the reference trace with a better quality to reduce noise enhancement upon division or try to fit the ih-RIDME traces without division. The latter option involves the risk of picking up RIDME artefacts in the fit. However, the influence on p(σ) is expected to be within the uncertainty range in the presence of substantial noise. In addition, using model distributions (e.g. mono-Gaussian and multi-Gaussian) is a way to stabilize the fitting further. As in the case of DEER data analysis [Bibr bib1.bibx45], the application of neural networks might also stabilize the solution.

#### 4.3.3 Determination of $D/\sigma^{3}$ and β

The optimization problem, as in Eq. ([Disp-formula Ch1.E25]), is parameterized by $D/\sigma^{3}$ and β besides the distribution p(σ) . Above, we discussed how these parameters determine the shape of the ih-RIDME kernel $K(\sigma t;T_{\text{mix}})$ : $D/\sigma^{3}$ regulates the dependence of the RIDME decay on mixing time and β determines the approximate shape of the F factor. These parameters are sensitive to the properties of an inhomogeneous proton distribution and, therefore, should be optimized for each dataset.

We performed a relaxed scan of $D/\sigma^{3}$ and β of a simulated dataset. For each combination of $D/\sigma^{3}$ and β , the pre-generated dataset was fitted with a model-free distribution with the same number of optimization steps. The analysis of the resulting 2D root-mean-square deviation (RMSD) plot (Fig. [Fig Ch1.F6]a and b) shows that $D/\sigma^{3}$ can be determined rather certainly (i.e. the 1D section through the optimal point demonstrates high curvature at the minimum). It turns out that incorrect values of $D/\sigma^{3}$ have characteristic manifestations in the time-domain fits (Fig. [Fig Ch1.F6]c). At lower values, the fit outputs an “extended” fork. Conversely, with the overestimated $D/\sigma^{3}$ , the typical “shrunk” fork is found in the time domain.

In contrast, parameter β is characterized by larger uncertainties due to the trace division step. For a homogeneous system, i.e. for $p(\sigma )=\delta (\sigma -\sigma_{0})$ for some $\sigma_{0}$ , the F factor cancels exactly, making β strictly unidentifiable from the reference-divided data. Broad distributions p(σ) lead to a weak dependence of the computed reference-divided dataset on β , as in Fig. [Fig Ch1.F6]b (green line). For water–glycerol glass in [Bibr bib1.bibx28], we found β≈0.13 , which can be used as an initial guess in the data analysis.

**Figure 6 Ch1.F6:**
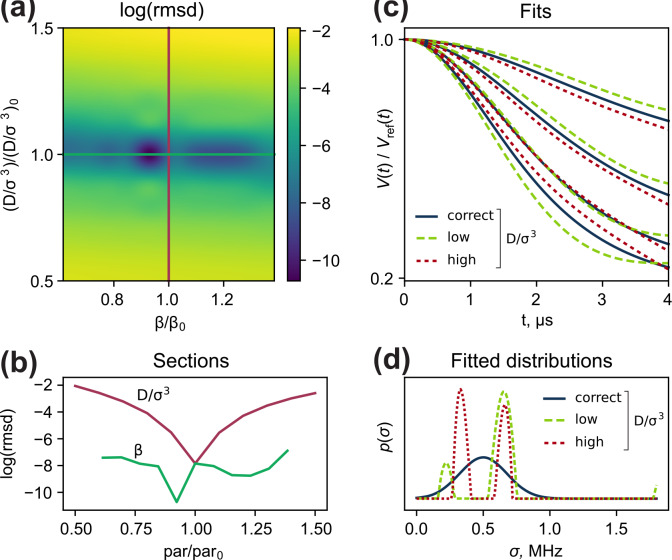
**(a)** Relaxed scan of $D/\sigma^{3}$ and β . The axes correspond to the deviations of the scanned parameters from those used for the dataset generation ( $(D/\sigma^{3}{)}_{0}$ and $\beta_{0}$ ). **(b)** Sections of the RMSD plot in panel **(a)** where one of the parameters is fixed. **(c)** Fitting results in the time domain with a fixed $\left(D/\sigma^{3}\right)$ of low (dashed green line), optimal (solid navy line) and high (dotted red line) values – $0.5(D/\sigma^{3}{)}_{0}$ , $(D/\sigma^{3}{)}_{0}$ and $1.5(D/\sigma^{3}{)}_{0}$ , respectively. **(d)** Corresponding fitted local proton concentration distributions.

### 4.4 Test on model compound 1

#### 4.4.1 The ih-RIDME study

We demonstrate the applicability of the above principles on model compound 1. This compound contains the same nitroxide unit as the widely used methanethiosulfonate spin label (MTSL). This molecule is also characterized by an anisotropic distribution of protons with respect to the electron spin, as is the case in MTSL-labelled proteins. The chemical structure of compound 1 is shown in Fig. [Fig Ch1.F7]a. It contains a shape-persistent linear backbone of conjugated benzene rings and ethynylene units. Seven polyethylene glycol (PEG) chains are attached to this backbone. These chains are conformationally highly flexible, due to essentially unhindered rotations around C–C and C–O bonds, and show no order. The two ethynylene units allow unconstrained dihedral angles between the planes of the benzene rings such that the radical consists of three sections that can freely rotate with respect to each other. Overall, we expect a broad conformational ensemble for this radical.

**Figure 7 Ch1.F7:**
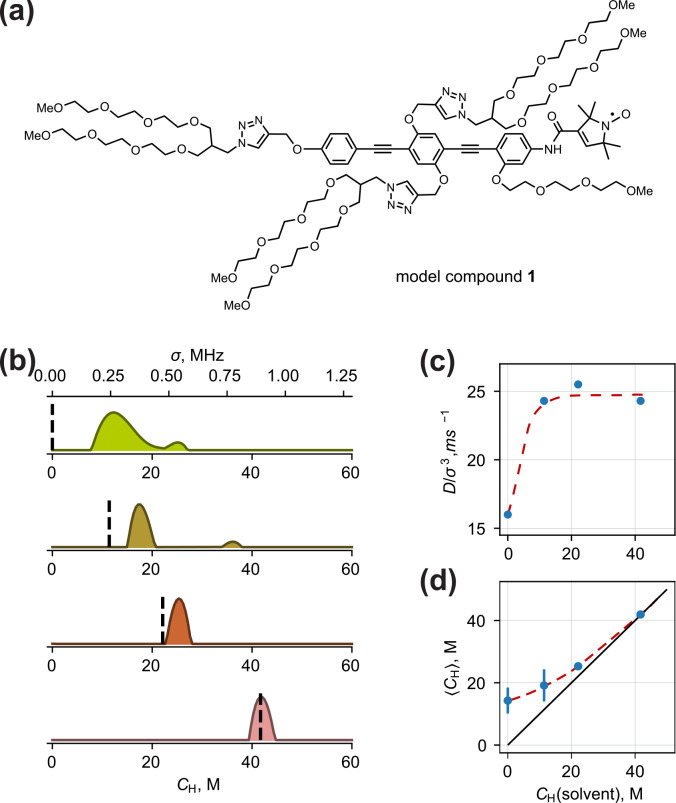
**(a)** Chemical structure of model compound 1 for testing solvent contrast. **(b)** Fitted model-free proton density distributions of compound 1 dissolved in $H_{2}O$ – $D_{2}O$ – $D_{8}$ -glycerol of different protonation degrees. The distributions from top to bottom correspond to the rows in Table [Table Ch1.T1]. Dashed lines mark the proton concentration of the solvent. **(c)** The optimal values of $D/\sigma^{3}$ depending on the solvent proton concentration. **(d)** Correlation between the solvent proton concentration and the mean proton concentration from ih-RIDME ( $\langle C_{\text{H}}\rangle$ ). The length of the vertical lines is equal to the standard deviation of the fitted distributions. Solvent deuteration unveils the internal protons of the solute as the dots deviate from the diagonal line. The dashed lines in panels **(c)** and **(d)** show the trend in the parameter change.

The fitted model-free distributions are presented in Fig. [Fig Ch1.F7]b. There, both $C_{H}$ and σ axes are given with a conversion coefficient of 0.0215 MHz M^−1^. The dashed vertical lines mark the proton concentration of the solvents in each sample. We observe that, in fully deuterated solvent, the distribution of proton densities represents relatively high $C_{H}$ values (between 10 and 20 M). This reflects that ih-RIDME decay is dominated by the hyperfine interaction with protons of the molecule (PEG side chains, benzene and triazene rings). The obtained distribution is relatively broad, which confirms our expectations from the analysis of the radical geometry.

When the solvent protonation is elevated, we observe two main changes in the p(σ) , namely, a shift in the mean value and narrowing. The former effect is explained by the increasing number of protons around the electron spin. When the solvent proton density matches the solute proton density, the expected value of p(σ) matches the solvent proton concentration. This is depicted in the correlation diagram in Fig. [Fig Ch1.F7]d. The deviation of the low- $C_{H}$ branch from the diagonal due to the protons of the solute is called the solvent contrast effect. The influence of the solvent isotope composition on the distribution width is also a part of this effect. This means that the effective anisotropy of the spatial proton distribution is maximal in the absence of solvent protons.

In practice, full solvent deuteration is not always possible, e.g. if a protonated stock solution is used for a dilution. The solvent protonation degree at which the solvent contrast effect disappears depends on the volume of the studied solute, i.e. on how many solvent molecules are displaced by the (macro)molecule. For a relatively small molecule like compound 1, this threshold apparently lies below $C_{H}=10$ mol L^−1^. For larger solutes, like proteins, protein complexes or biomolecular condensates, characterization of the solute may be possible at higher residual protonation of the solvent.

We also note a solvent effect of a different kind. The optimal $D/\sigma^{3}$ was found at 16 ms^−1^ in a fully deuterated medium and at 24–26 ms^−1^ in partially protonated samples (Fig. [Fig Ch1.F7]c). We connect this to the impact of solvent protons on the connectivity of the proton bath. The main source of protons in compound 1 is the PEG chains. It is plausible to assume that, due to the conformational flexibility and weak interchain interaction, the PEG chains have, at most, a few contacts (with each other) per chain in an average conformer. This means that all $(-{\mathrm{CH}}_{2}-{\mathrm{CH}}_{2}-O-{)}_{n}$ chains can be seen as approximately isolated (i.e. an average interchain distance is large, and the coupling between protons belonging to different chains can be neglected). Therefore, the homonuclear flip-flops within the same PEG chain predominate. Solvent protons, being homogeneously distributed, mediate nuclear spin diffusion between the chains and, thus, enhance spectral diffusion. In particular, the spectral diffusion coefficient D is expected to grow in such a proton configuration compared with the configuration with a quasi-isolated group of protons with a similar value of σ . Accordingly, the ratio $D/\sigma^{3}$ is higher. This model agrees with the estimated average interproton distance in water–glycerol solvent, which is $r_{H,H}=5$ Å for $C_{H}=10$ M. Such a distance is smaller than the length of the PEG chain; therefore, the chains can indeed magnetically interact with the solvent protons. Further increase in the proton concentration does not have an effect. Consequently, the value of $D/\sigma^{3}$ may give information on solvent accessibility or interchain contacts of parts of a molecule, substantially separated from the electron spin.

#### 4.4.2 In silico analysis

We performed in silico analysis of the p(σ) distribution. We generated an unrestrained conformational ensemble of model compound 1 using the Monte Carlo (MC) approach. For the generation, we used the dihedral angle potentials from [Bibr bib1.bibx14]. More details on the ensemble generation are given in Supplement S6. The ensemble included 1500 conformers.

The generated MC ensemble (Fig. [Fig Ch1.F8]a) confirmed a significantly anisotropic distribution of the protons. They are concentrated on one side of the nitroxide fragment. This means that molecules with a different orientation in the external magnetic field have different hyperfine spectra (as discussed in Sect. [Sec Ch1.S4.SS1]) and that orientation averaging must be done. Model compound 1 contains 158 chemically unexchangeable protons, 13 of which belong to the rigid nitroxide unit. The remaining 145 protons are distributed within a volume of approximately 15 ${\mathrm{nm}}^{3}$ if the boundaries of the conformational ensemble are approximated as a box. This results in an estimated mean local proton concentration of 16.1 M, which is in good agreement with the ih-RIDME data. Nevertheless, the protons are distributed inhomogeneously and form distinct clouds localized around the PEG chains. On the larger scale, the proton ensemble has an ellipsoidal shape with a maximal electron–proton distance of ca. 3.5 nm.

We converted this ensemble into a distribution of local proton densities. For each conformer, we computed the hyperfine coupling constants with protons in point-dipole approximation. The width of the corresponding hyperfine spectrum σ was calculated according to Eq. ([Disp-formula Ch1.E10]). The averaging of the molecular orientation was done by sampling the direction of the external magnetic field (and, thus, the electron's and nuclear spin's quantization axis) over a spherical grid.

To reproduce the experimental distribution p(σ) , it is important to determine which protons of the molecule contribute to the spectral diffusion in the ih-RIDME experiment. We performed a simple estimation of the spin diffusion radius by introducing the global cutoff radius $r_{\mathrm{cut}}$ . The protons lying closer to the electron spin than the cutoff are excluded from the nuclear ensemble. We scanned $r_{\mathrm{cut}}$ in the range from 0.5 to 2.0 nm with a step of 0.05 nm and recomputed the distribution of σ at each value (see Fig. [Fig Ch1.F8]b). If close protons are included in the calculation, the predicted distribution is shifted to the higher mean values and is broadened. We found that $r_{\mathrm{cut}}=1.55$ nm leads to the best agreement of the distribution functions based on the RMSD criterion. At this value, the mean values of the experimental and calculated distributions are also close (see Supplement S6). The comparison of the standard deviation and the skewness shows a deviation, and the reason for this can be found in both the experimental uncertainties and the ensemble generation. Overall, we conclude that the unrestrained conformational ensemble of compound 1 satisfactorily reproduces the experiment. This could be expected because model compound 1 does not contain groups with specific interaction, e.g. hydrogen bonding. We tried to improve the agreement by choosing a different force field or by allowing the distribution around the canonical dihedral angles. It was found that these steps had a weak influence on the properties of the conformational ensemble and, accordingly, a weak effect on the predicted distribution of local proton density.

A further improvement step would be to develop a better physical model for the spin diffusion radius and for blocked spins based on the nuclear coordinates. Here, we used a spherical-surface approximation to mark the inactive nuclei. Thus, our presented calculation in this approximation provides an understanding of the sensitivity range of the ih-RIDME method. In reality, due to the anisotropy of the hyperfine and nuclear dipolar interactions, the blocking surface may be non-spherical [Bibr bib1.bibx7]. With the development of methods to analyse the behaviour of multi-nuclear spin systems, it would be possible to carry out more accurate structure-based predictions of the active zone in ih-RIDME. For example, it is feasible to expect that the characteristic size of the spin diffusion radius may be different for the conformers. Furthermore, in relatively compact structures with high anisotropy, where electron–nuclear vectors are significantly correlated with nuclear–nuclear vectors, the diffusion-active zone may also depend on the orientation of the molecule in the external magnetic field. In addition, there is evidence that the nuclear spin dynamics is not entirely frozen in the blocked volume [Bibr bib1.bibx43]. Thus, the nuclei below $R_{\text{sd}}$ may contribute to the electron spectral diffusion. However, observation of this requires using waiting times in RIDME above tens of milliseconds, and this would only be possible at temperatures lower than 50 K due to the electron $T_{1}$ limitation.

**Figure 8 Ch1.F8:**
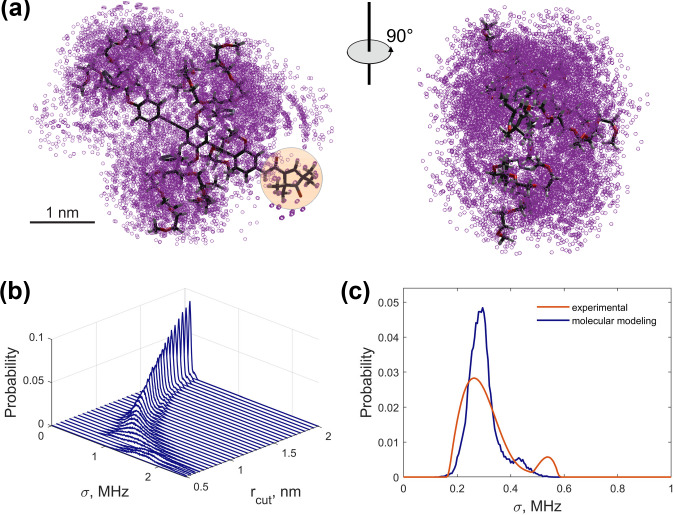
In silico analysis of the MC-generated conformational ensemble of compound 1: **(a)** a 3D structure and ensemble of 100 randomly selected conformers aligned by the rigid backbone. Magenta circles represent the positions of protons. The nitroxide fragment is highlighted in orange. **(b)** Calculated distributions p(σ) for different cutoffs. **(c)** The experimental distribution p(σ) (orange) and the computed distribution with $r_{\text{cut}}=1.55\;\mathrm{nm}$ . The visualization of the cutoff radius in the molecular ensemble can be found in Fig. S16 (Supplement S6).

### 4.5 Comparison of ih-RIDME sequences

The 5p-RIDME experiment that has been thoroughly investigated so far has some disadvantages and limitations. First of all, the target echo in 5p-RIDME is separated from the beginning of the experiment by a long time, of which ( $2d_{1}+2d_{2}$ ) is the transverse evolution that can reach values of 10 µ s or even more. Due to this, the echo intensity is additionally reduced. Our numeric tests showed that the noise leads to large uncertainties in the distribution shape. Thus, a long measurement time is needed to accumulate the necessary SNR. Moreover, if the ih-RIDME method is applied to systems with a substantially broad proton density distribution, the high-concentration fraction may appear attenuated in the experimental data due to $d_{2}$ filtering. As an extreme, if the $d_{2}$ delay exceeds the phase memory time of some spin packets by a large factor, these spin packets will not contribute to the ih-RIDME signal and will not be represented in the distribution. On the other hand, short delays $d_{2}$ limit the resolution of the low-concentration part, as was demonstrated above. This problem is also discussed for 4p-DEER, which is also a constant-time experiment [Bibr bib1.bibx19], and from the application side of RIDME in PDS [Bibr bib1.bibx46]. The common principle to reduce or avoid the filtering effects is the use of variable-time experiments.

**Figure 9 Ch1.F9:**
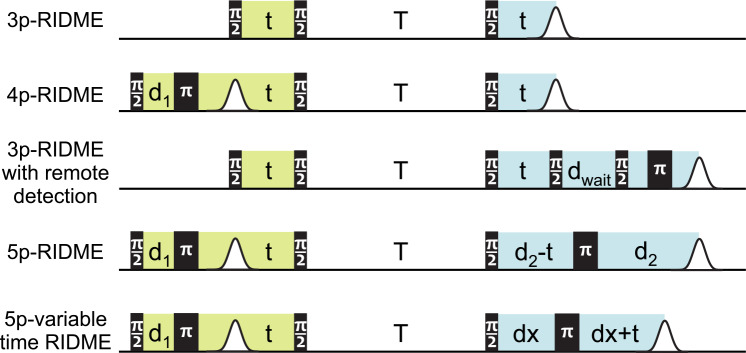
Variants of ih-RIDME pulse sequences. In all experiments, the delay t is incremented. The position of the echo in 5p-RIDME is constant, and it shifts in all other experiments with the step 2Δt , where Δt is the increment step of the delay t . The lime and the blue colours highlight the respective preparation and the detection parts of the sequences (see Fig. [Fig Ch1.F1]). The phase-cycling protocols for the shown sequences are gathered in Appendix [App App1.Ch1.S3].

We consider four possible analogues presented in Fig. [Fig Ch1.F9] and compare them with 5p-RIDME. These sequences have a common mixing block and differ with respect to the preparation and the detection blocks. In 5p-RIDME, the fixed delay $d_{2}$ limits the available evolution time. In the 3p-RIDME experiment ([Bibr bib1.bibx26]; the first sequence in Fig. [Fig Ch1.F9]), the traces may be recorded with an arbitrary length (i.e. there is no upper limit on the value of delay t imposed by the pulse sequence). The 3p-RIDME experiment, however, also has disadvantages. First, the three-pulse sequence does not provide an option for ${}^{2}H$ -ESEEM averaging. Nevertheless, we observed experimentally on various systems that the nuclear modulation cancels out after the division by the reference trace [Bibr bib1.bibx34]. We suggest using the 4p-RIDME sequence (second sequence in Fig. [Fig Ch1.F9]), in which the ESEEM averaging is achieved by variation in the first interpulse delay, similar to the standard 5p-RIDME ([Bibr bib1.bibx22]; see Supplement S7 for details). Another important issue is the spectrometer dead time that prevents data acquisition at short delays t ( t<200ns for Q-band spectrometers). Due to this, additional uncertainties in the trace normalization can be anticipated. This problem is mitigated by remote detection (3pRD-RIDME; the third sequence in Fig. [Fig Ch1.F9]). The principle is to apply a microwave (mw) pulse with an orthogonal phase at the centre of the echo and, thus, rotate the magnetization vector to the z axis (i.e. convert coherence to polarization). The length of the magnetization vector does not change and is read out by the last two pulses. The longitudinal waiting time $d_{\text{wait}}$ needs to exceed the spectrometer dead time but should be short enough so that electron longitudinal relaxation is negligible. The experimental traces of 3pRD-RIDME were measured with $d_{\text{wait}}=1$ µ s. The delay in the read-out block may be as short as is allowed by the instrumentation. The remaining dead time, in this case, is determined by the properties of the mw amplifier, namely, the minimal separation time when pulses do not interact with each other. The travelling-wave tube (TWT) amplifier, available to us, allowed for a pulse separation of 32 ns . We also demonstrate experimentally (see Fig. S17 in Supplement S7), by comparing 3p-RIDME and 3pRD-RIDME, that the remote detection block does not distort the ih-RIDME data. As the trace decay in ih-RIDME is usually slower than in PDS techniques, the dead-time reconstruction should be more reliable in this case and can be achieved by extrapolation. We also considered a variable-time RIDME sequence proposed in [Bibr bib1.bibx46] (fifth sequence in Fig. [Fig Ch1.F9]). The detection principle exploits a single π pulse to refocus the virtual echo. The delay dx is fixed and should be chosen to be larger than $d_{1}$ due to a crossing echo that is not eliminated by a standard eight-step phase-cycling protocol. The advantage of this experiment is that it provides both dead-time-free measurement and an option for ESEEM averaging. We refer to this experiment as 5pVT-RIDME.

**Figure 10 Ch1.F10:**
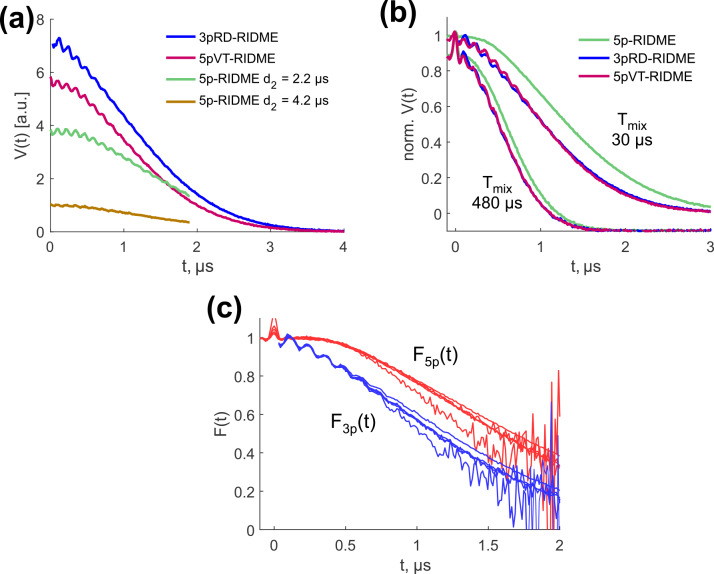
**(a)** Comparison of absolute sensitivity in 3pRD-RIDME (blue), 5pVT-RIDME (red), 5p-RIDME with $d_{2}=2.2$ µ s (green) and $d_{2}=4.2$ µ s (yellow). The mixing time was set to 15 µ s. **(b)** Comparison of the trace curvature in 5p-RIDME (green), 3pRD-RIDME (blue) and 5pVT-RIDME (red) at $T_{\text{mix}}=30$ µ s and $T_{\text{mix}}=480$ µ s (vertically shifted for better visibility). **(c)** Transverse factors extracted from 3pRD-RIDME (blue) and 5p-RIDME (red).

Next, we compare the sensitivity of 3pRD-RIDME, 5pVT-RIDME and 5p-RIDME. For this, we measured the corresponding traces with the same microwave setup and number of shots per point. Thus, the level of noise is comparable between the datasets and the comparison of signal intensity represents the comparison of signal-to-noise ratios. For the tests, we measured the 5p-RIDME signal with $d_{2}$ parameters equal to 2.2 and 4.2 µ s. The results are presented in Fig. [Fig Ch1.F10]a. We found that the 3pRD-RIDME signal close to t=0 exceeds both 5p-RIDME signals. The sensitivity enhancement was calculated at t=0.08 µ s and yielded 1.9 for $d_{2}=2.2$ µ s (green line) and 7.2 for $d_{2}=4.2$ µ s (yellow line). These values correspond to the reduction in the measurement time by a factor of ≈3.5 and ≈52 , correspondingly. The major contribution to such an enhancement stems from the decrease in the total transverse evolution time. However, less magnetization loss due to fewer pulses in 3p-RIDME also plays a role. It is discussed theoretically in [Bibr bib1.bibx38] and shown numerically in [Bibr bib1.bibx31] that the total excitation profile of a pulse sequence is narrower with an increased number of mw pulses. Consequently, fewer spin packets contribute and the signal intensity is lower. The slight intensity reduction in 5pVT-RIDME compared with 3pRD-RIDME (by factor of 1.3 under the conditions of Fig. [Fig Ch1.F10]a) is most probably explained by the longer total transverse evolution time in 5pVT-RIDME compared with 3pRD-RIDME.

Next, we discuss the data model of the ih-RIDME experiment's signal. All sequences in Fig. [Fig Ch1.F9] can be split into the preparation, spectral diffusion and detection parts, as was done for 5p-RIDME. Following the spin dynamics treatment summarized in Sect. 2, we know that the ih-RIDME signal is a product of a longitudinal spectral diffusion factor $R(t;T_{\text{mix}})$ , which is independent of the type of the pulse sequence, and the factor F(t) , which is determined by the preparation and detection blocks in a specific pulse sequence:

29 $$V\left(t;T_{\text{mix}},d\right)\approx R\left(t;T_{\text{mix}}\right)\cdot F\left(t;d\right),$$

where d denotes an array of all constant auxiliary delays specific to the chosen pulse sequence. For 5p-RIDME, $d=[d_{1},d_{2}]$ ; for 5pVT-RIDME, $d=[d_{1},\mathrm{dx}]$ ; etc. At the mixing times, which validate the approximation (Eq. [Disp-formula Ch1.E29]), the spin dynamics in the preparation and the detection blocks are uncorrelated, and the transverse factor is additionally factorized [Bibr bib1.bibx30]:

30 $$F(t;d)=F_{\text{prep}}\left(t;d_{\text{prep}}\right)\cdot F_{\text{det}}\left(t;d_{\text{det}}\right)\;.$$

The experimental 3pRD-RIDME and 5pVT-RIDME traces are characterized by a faster decay than 5p-RIDME traces, as can be seen from superimposed normalized data in Fig. [Fig Ch1.F10]b. As the longitudinal factor is common in all three experiments, the transverse factor determines the difference. Consequently, the transverse decay $F_{3p}(t)$ is steeper than $F_{5p}(t)$ . In Fig. [Fig Ch1.F10]c, we compared them directly by extracting from the experimental traces (see Supplement S7 for details). A Gaussian fit of $F_{3p}(t)$ resulted in β≈0.40 . Slower decay of $F_{5p}(t)$ may be attributed to the role of the last π pulse in 5p-RIDME. It partially refocuses the electron–nuclear interactions and enables a constant transverse evolution time, which balances the decay of $F_{5p}(t)$ . The faster decay of $F_{3p}(t)$ and $F_{5\mathrm{pVT}}(t)$ , in turn, may cause a significantly non-uniform SNR in the reference-divided traces that decreases fast towards the end of the traces. In principle, new ih-RIDME sequences may be designed by modifying the preparation and detection parts to optimize the properties of the transverse factor.

Based on the presented comparison, we conclude that 3p-RIDME and 5pVT-RIDME outperform the constant-time 5p-RIDME and, therefore, can be considered preferable experiments for the ih-RIDME study. In PDS, the steepness of the background should be minimized to facilitate its separation from the electron–electron dipolar form factor. This is usually achieved in pulse experiments with a static observer sequence. The variable-time experiments feature additional electron–nuclear decay, which is why they are less popular in PDS. The signal in ih-RIDME is already determined by the electron–nuclear interactions; therefore, changing from a constant-time experiment to a variable-time experiment has a weaker effect on the steepness of the trace decay.

All presented sequences contain a longitudinal block and can be used for the RIDME experiment to detect the electron–electron dipolar interaction. We see potential with respect to developing and designing the pulse sequences with longitudinal blocks in the hyperfine spectral diffusion phenomenon context. As proposed above, the optimization approach can deal with changing the preparation and detection part. Moreover, in principle, the use of multiple mixing blocks can be also investigated. Therefore, we propose HYperfine Spectral Diffusion Echo MOdulatioN (HYSDEMON) as an alternative name for this series of experiments that emphasizes the mechanism dominating the signal generation.

## 5 Conclusions

In the present work, the fundamentals for the analysis of the heterogeneous nuclear ensembles using the ih-RIDME method are considered.

The electron–proton contribution to the electron spin echo decay in the RIDME experiment can be rather well approximated as a result of spectral diffusion of the electron spin within a Gaussian-shaped distribution of the hyperfine field. Accordingly, the width σ of such a Gaussian distribution is a measure of the number of protons in the vicinity of the electron spin as well as of the mean distance from the electron spin to the proton cloud. The normalized diffusion coefficient $D/\sigma^{3}$ in such a description becomes a universal parameter which characterizes the connectivity within the proton cloud and is independent of the position and shape of this cloud. For homogeneous frozen solvent glasses, fitting the ih-RIDME data allows one to detect differences between the characteristic hyperfine spectrum width and characteristic electron spectral diffusion rate between different types of glasses.

RIDME data are recorded as a series of decays corresponding to different mixing times and fitted in a reference-divided form. Such global fitting of multiple traces substantially stabilizes the output of RIDME data analysis. Due to the stability of RIDME data fitting, it is also possible to analyse data for samples with varying densities of protons in the vicinity of paramagnetic centres. The ih-RIDME data are fitted assuming a distribution of σ values. Such inhomogeneous proton density distributions correlate with the statistics of interchain contacts of unfolded biopolymers. Thus, the distribution functions can then be converted to structural information.

We investigated and characterized the numeric properties of ih-RIDME data fitting, including uncertainties in the distribution's mean and standard deviation, robustness to noise, and determination of the parameters of the ih-RIDME kernel. The main results are that the mean value is a reliable output of the fitting routine under a broad range of conditions and that the determination of the distribution's shape features may require optimization of the lengths and the signal-to-noise ratios of the datasets.

These results were further applied to the study of a model compound with a nitroxide, as a source of the electron spin, and with conformational highly flexible proton-rich fragments and a substantially anisotropic proton distribution. In a fully deuterated solvent, in the approximation of the rigid cutoff, a spin diffusion radius of 1.55 nm was found. With this radical, we demonstrated two kinds of solvent protonation effects, namely, masking the heterogeneity of the local proton environment and enhancement of the spectral diffusion kinetics mediated by the solvent.

Finally, we considered alternative pulse sequences for the ih-RIDME experiment. While the standard constant-time five-pulse RIDME experiment can introduce additional phase-memory filtering of the proton density distribution function, the three-pulse RIDME and variable-time five-pulse RIDME are free of this effect. In addition, they provide a higher signal-to-noise ratio and, thus, can be considered to be preferential sequences for ih-RIDME.

## Supplement

10.5194/mr-6-93-2025-supplement The supplement related to this article is available online at https://doi.org/10.5194/mr-6-93-2025-supplement.

## Supplement

10.5194/mr-6-93-2025-supplement
10.5194/mr-6-93-2025-supplement The supplement related to this article is available online at https://doi.org/10.5194/mr-6-93-2025-supplement.

## Figures and Tables

**Figure A1 App1.Ch1.S1.F11:**
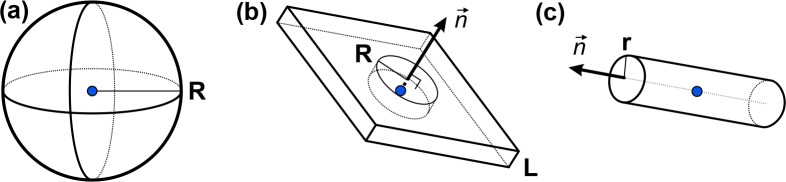
**(a)** Spherical (3D), **(b)** planar (quasi-2D) and **(c)** cylindrical (quasi-1D) distributions of nuclei. The illustrated parameters R , n , L and r are used in Eqs. ([Disp-formula App1.Ch1.S1.E38])–([Disp-formula App1.Ch1.S1.E40]). The blue point marks the position of the electron spin.

## Data Availability

The experimental data, including the EPR and NMR data, are available online at Zenodo: 10.5281/zenodo.14017046 [Bibr bib1.bibx27].

## Appendix A The width of the hyperfine spectrum in a proton environment of low dimensionality

In the main text, we show that the variance of the hyperfine spectrum of N nuclei of spin I is expressed via the hyperfine coupling constants as follows:

A1
$$\sigma^{2}=\frac{I(I+1)}{3}\sum_{j=1}^{N}A_{j}^{2}\;.$$

We can use the point-dipole approximation for the hyperfine interaction and apply the following:

A2
$$A_{j}=\frac{\mu_{0}\hbar \gamma_{e}\gamma_{n}}{4\pi }\frac{1-3{\mathrm{cos}}^{2}\theta_{j}}{r_{j}^{3}},$$

where $\gamma_{e}$ and $\gamma_{n}$ are the electron and nuclear gyromagnetic ratios, respectively; $r_{j}$ is the electron–nuclear distance; and $\theta_{j}$ is the dipolar angle. Substitution into Eq. ([Disp-formula App1.Ch1.S1.E31]) gives the following:

A3
$$\sigma^{2}=\frac{I(I+1)}{3}\cdot {\left(\frac{\mu_{0}\hbar \gamma_{e}\gamma_{n}}{4\pi }\right)}^{2}\sum_{j=1}^{N}\frac{(1-3{\mathrm{cos}}^{2}\theta_{j}{)}^{2}}{r_{j}^{6}}\;.$$

Summation in this formula can be replaced by integration over the volume V containing the nuclei if we introduce a nuclear density function ρ(r) :

A4
$$\begin{array}{rl} \sigma^{2}(V)= & \frac{I(I+1)}{3}\cdot {\left(\frac{\mu_{0}\hbar \gamma_{e}\gamma_{n}}{4\pi }\right)}^{2}\\ & \int_{V}\frac{(1-3{\mathrm{cos}}^{2}\theta {)}^{2}}{r^{6}}\rho (r)dV\;. \end{array}$$

In a model computation, we choose the volume of interest as a spherical shell with an inner radius $R_{1}$ and outer radius $R_{2}$ centred at the electron spin. In addition, we assume a spherically symmetric approximation for ρ(r) which (in spherical coordinates) reads as follows:

A5
$$\rho (r)=4\pi r^{2}g(r)C_{\text{H}},$$

where g(r) is radial distribution function and $C_{\text{H}}$ is proton number density. Taking, for simplicity, g(r)=1 , we obtain the expression in spherical coordinates:

A6
$$\begin{array}{rl} \sigma^{2}\left(R_{1},R_{2}\right) & =\frac{I(I+1)}{3}\cdot {\left(\frac{\mu_{0}\hbar \gamma_{e}\gamma_{n}}{4\pi }\right)}^{2}\int_{R_{1}}^{R_{2}}dr\frac{4\pi r^{2}}{r^{6}}C_{H}\\ & \times \int_{0}^{\pi }{\left(1-3{\mathrm{cos}}^{2}\theta \right)}^{2}\frac{\mathrm{sin}\theta }{2}d\theta \;. \end{array}$$

The inner integral equals four-fifths; therefore, the final value is as follows:

A7
$$\sigma^{2}(R_{1},R_{2})=\frac{4\pi }{3}B^{2}C_{\text{H}}\left(\frac{1}{R_{1}^{3}}-\frac{1}{R_{2}^{3}}\right),$$

where $B=|\hbar \mu_{0}\gamma_{e}\gamma_{n}/4\pi |\cdot (4I(I+1)/15{)}^{1/2}$ . If the upper bound is at infinite distance, $R_{2}=+\infty$ , we obtain the following:

A8
$$\sigma^{2}(R_{1},\infty )=\frac{4\pi }{3}B^{2}\frac{C_{\text{H}}}{R_{1}^{3}},$$

which reproduces Eq. ([Disp-formula Ch1.E11]).

We performed similar computations for different model distributions of lower local dimensionality. We used Eq. ([Disp-formula App1.Ch1.S1.E34]) with changed density functions ρ(r) . For instance, a spin-labelled membrane protein in a protonated membrane in a deuterated solvent can effectively experience a quasi-2D proton distribution. The computation of the variance of the hyperfine spectrum is, in this case ( R≫L ), as follows:

A9
$$\sigma^{2}(R,\infty )=\frac{5\pi }{8}\left(1-3n_{z}^{2}+\frac{27}{8}n_{z}^{4}\right)LB^{2}\frac{C_{H}}{R^{4}}\;,$$

where $n_{z}$ is the z component of the plane's normal vector (Fig. [Fig App1.Ch1.S1.F11]) and B is defined as in Eq. ([Disp-formula Ch1.E11]). $C_{H}$ in the equation above is the proton density in the bi-layer. Repeating the same calculations for quasi-1D geometry, i.e. a cylinder of radius r and of the symmetry axis $n=(n_{x},n_{y},n_{z}{)}^{T}$ , yields the following:

A10
$$\sigma^{2}(R,\infty )=\frac{2\pi }{5}{\left(1-3n_{z}^{2}\right)}^{2}r^{2}B^{2}\frac{C_{H}}{R^{5}}\;.$$

The 1D and 2D geometries are characterized by a steeper dependence on R than the 3D case. Distant nuclei have a smaller contribution to the hyperfine spectrum, and variation in their positions has weaker impact on σ . Consequently, we expect that the sensitivity range in the ih-RIDME method is closer to the electron spin in lower dimensions compared with the 3D distribution.

## Appendix B Derivation of Eq. (19)

We start from Eq. ([Disp-formula Ch1.E11]) for a uniform 3D nuclear distribution assuming nuclear density $C_{n}$ and nuclear magnetic moment $\mu_{\text{nuc}}$ :

B1
$$\sigma_{R}=\sqrt{\sigma^{2}(\infty )-\sigma^{2}(R)}\propto |\mu_{\text{nuc}}|\sqrt{\frac{C_{n}}{R^{3}}}\;.$$

The cutoff radius $R_{\text{sd}}$ is approximately determined by the condition that the difference in the hyperfine coupling between the neighbouring nuclei is comparable with their dipolar interaction $|A_{j}-A_{k}|\approx \omega_{j,k}$ . To obtain qualitative relations, we neglect the angular dependence of both hyperfine and nuclear dipolar interactions. By denoting the minimal distance between the nuclei of the same sort as $d_{\text{min}}$ , we obtain the following:

B2
$$\frac{\mu_{0}}{4\pi \hbar }\frac{3|g_{e}\mu_{B}\mu_{\text{nuc}}|}{R_{\text{sd}}^{4}}d_{\text{min}}\approx \frac{\mu_{0}}{4\pi \hbar }\frac{\mu_{\text{nuc}}^{2}}{d_{\text{min}}^{3}},$$

where the expression on the left-hand side is the first-order Taylor expansion with respect to the $d_{\text{min}}$ . Solving for $R_{\text{sd}}$ , we find the following:

B3
$$R_{\text{sd}}\approx \sqrt[{4}]{3\left|\frac{g_{e}\mu_{B}}{\mu_{\text{nuc}}}\right|}d_{\text{min}}\;.$$

In a uniform nuclear bath $d_{\text{min}}\propto C_{n}^{-1/3}$ ,

B4
$$R_{\text{sd}}\propto |\mu_{\text{nuc}}{|}^{-1/4}C_{n}^{-1/3}\;.$$

After substitution of $R_{\text{sd}}$ into Eq. ([Disp-formula App1.Ch1.S2.E41]), Eq. ([Disp-formula Ch1.E19]) is derived.

## Appendix C Phase-cycling protocols for ih-RIDME experiments

In the following tables, the pulse index (p1, p2, etc.) corresponds to the pulse order in Fig. [Fig Ch1.F9]. The phases of the unmentioned pulses are +x . The detection (det) denotes the sign of the echo acquisition.

**Table C1:** Phase-cycling protocol for three-pulse RIDME.

3p-RIDME			
p1	p2	p3	det
+x	+x	+x	+
-x	+x	+x	-

**Table C2:** Phase-cycling protocol for three-pulse RIDME with remote detection.

3pRD-RIDME			
p2	p3	p4	det
+x	+x	+x	+
-x	-x	+x	+
+y	+y	+x	+
-y	-y	+x	+
+x	+x	-x	-
-x	-x	-x	-
+y	+y	-x	-
-y	-y	-x	-

**Table C3:** Phase-cycling protocol for four-pulse RIDME.

4p-RIDME			
p1	p3	p4	det
+x	+x	+x	+
+x	-x	-x	+
-x	+x	+x	-
-x	-x	-x	-

**Table C4:** Phase-cycling protocol for five-pulse RIDME and five-pulse variable-time RIDME.

5p-RIDME and 5pVT-RIDME			
p1	p3	p4	det
+x	+x	+x	+
+x	-x	-x	+
+x	+y	+y	+
+x	-y	-y	+
-x	+x	+x	-
-x	-x	-x	-
-x	+y	+y	-
-x	-y	-y	-

## Appendix

The supplement related to this article is available online at https://doi.org/10.5194/mr-6-93-2025-supplement.
