# Supplementary material for: ih-RIDME: a pulse EPR experiment to probe the heterogeneous nuclear environment
Source: Magn Reson (Gott). 2025 Mar 10;6(1):93–112. doi: 10.5194/mr-6-93-2025 (PMC12247092; doi:10.5194/mr-6-93-2025)
Supplement: The supplement related to this article is available online at https://doi.org/10.5194/mr-6-93-2025-supplement. [file mr-6-93-2025-supplement.zip › mr-6-93-2025-supplement-title-page.pdf]

Supplement of Magn. Reson., 6, 93–112, 2025
https://doi.org/10.5194/mr-6-93-2025-supplement
© Author(s) 2025. CC BY 4.0 License.

Supplement of

ih-RIDME: a pulse EPR experiment to probe the heterogeneous nuclear environment

Sergei Kuzin et al.

Correspondence to: Sergei Kuzin (skuzin@ethz.ch)

- mr-6-93-2025-supplement-title-page.pdf
- mr-2024-19_supplement_part_1.pdf
- mr-2024-19_supplement_part_2.pdf

The copyright of individual parts of the supplement might differ from the article licence.
