# Supplementary material for: ih-RIDME: a pulse EPR experiment to probe the heterogeneous nuclear environment
Source: Magn Reson (Gott). 2025 Mar 10;6(1):93–112. doi: 10.5194/mr-6-93-2025 (PMC12247092; doi:10.5194/mr-6-93-2025)
Supplement: The supplement related to this article is available online at https://doi.org/10.5194/mr-6-93-2025-supplement. [file mr-6-93-2025-supplement.zip › mr-2024-19_supplement_part_1.pdf]

Contents

S1 Synthesis of model compound 1

S1 Synthesis of model compound 1

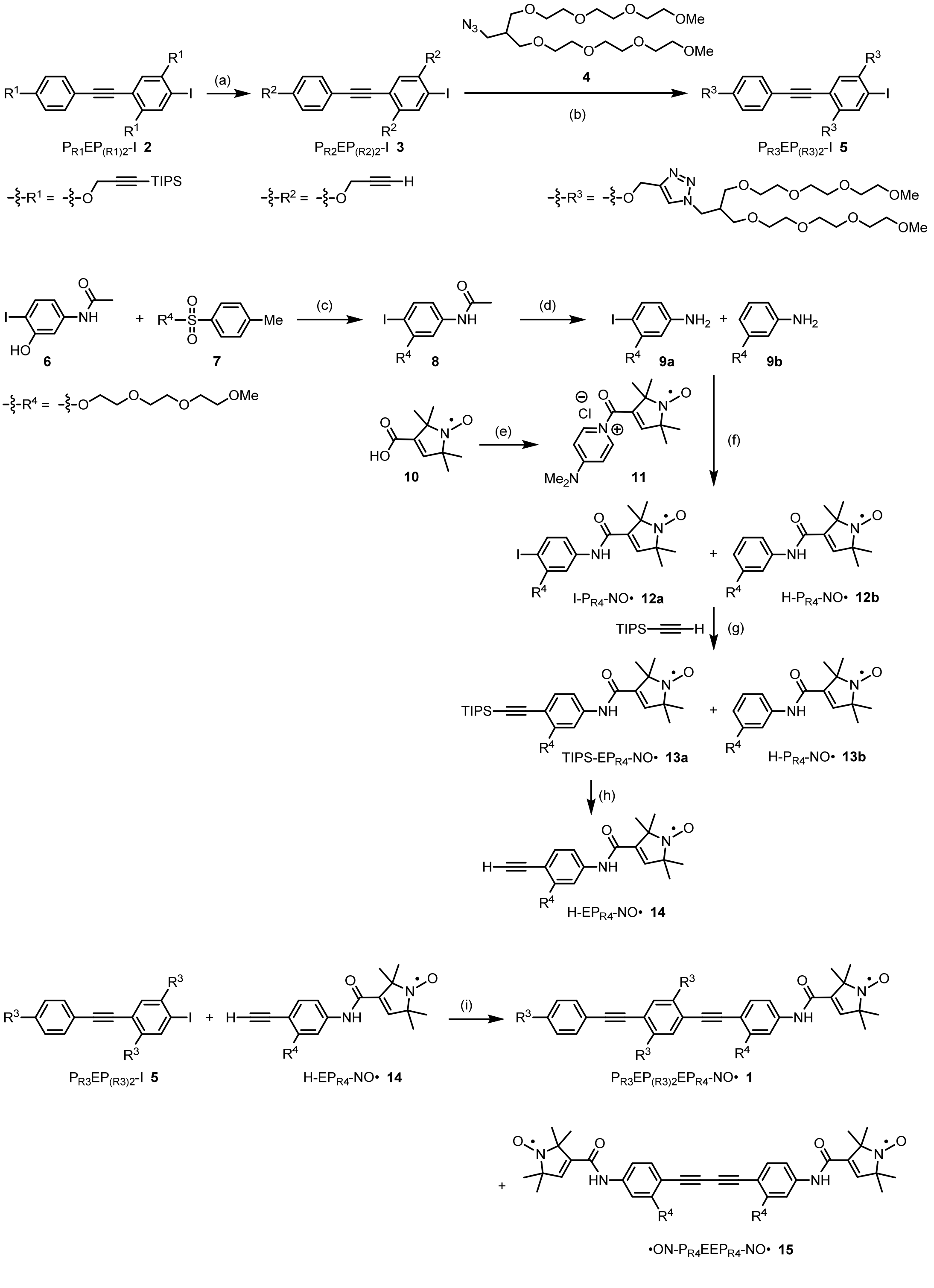

Scheme S1. Synthesis of model compound **1**. (a) $^{n}Bu_4NF$, THF, rt, 10 min; (b) $[Cu(phen)(PPh_3)_2]NO_3$•0.5 CH_2Cl_2, toluene, 40 °C, 50 h, 68% yield over two steps; (c) Na_2CO_3, KI, MeCN, 75 °C for 16 h then 90 °C for 44 h, 38%; (d) KOH, MeOH, 85 °C, 60 h, 61% yield of **9a** and 9% yield of **9b**; (e) $SOCl_2$, 4-(*N*,*N*-dimethylamino)pyridine, DMF, CH_2Cl_2, 0 °C → rt, 35 min; (f) CH_2Cl_2, 0 °C → rt, 16 h, 87% yield of **12a** and 94% yield of **12b**; (g) $PdCl_2(PPh_3)_2$, CuI, piperidine, THF, rt, 5 d, 36%; (h) (1) $^{n}Bu_4NF$, THF, rt, 10 min; (2) workup; (3) $^{n}Bu_4NF$, THF, rt, 16 h, 21%; (i) $Pd(PPh_3)_4$, $Pd_2(dba)_3$, CuI, piperidine, THF, rt, 20 h, yield of **1**: 45%; yield of **15**: 41%.

Nomenclature. In the experimental part, we use the following nomenclature to name the compounds (Scheme 1): The backbone building units *para*-phenylene and ethynylene units are abbreviated as P and E, respectively. R^1, R^2, R^3, and R^4 denominate the substituents at the benzene rings. P_{R1} represents a benzene ring with one substituent of type R^1, and $P_{(R1)2}$ indicates a benzene ring with two substituents of type R^1. NO• denominates the nitroxide moiety.

Experimental Part

General. Unless otherwise stated, reactions were performed using commercially obtained solvents and reagents. Dry toluene (99.85%, extra dry over molecular sieve, Acros Organics) and dry CH_2Cl_2 (99.8%, extra dry over molecular sieve, stabilized with approx. 50ppm amylene, Acros Organics) were used as received. $PdCl_2(PPh_3)_2$ was synthesized according to the literature.(Kukula et al., 1999) THF (HPLC, VWR) used in reactions were distilled from Na/benzophenone. Solvents used for extraction and chromatography were purchased in technical quality and distilled prior to use. Aqueous solutions were prepared using Milli-Q H_2O. Since all commercial compounds had a purity of >95%, their amount of substance given in the preparation procedures was calculated using their compound mass without correction through the manufacturer-specified purities.

Argon (Linde, 4.0) was passed through anhydrous $CaCl_2$ prior to use. Degassed solutions were obtained through three freeze−pump−thaw cycles. The temperatures given in the preparative procedures are the bath temperatures. Unless otherwise stated, solvents were removed at ~40 °C and reduced pressure using a rotary evaporator. Traces of remaining solvents were removed at room temperature at <1 mbar. For short path distillations, the reaction flask and the receiver flask were

connected by a short, curved glass tube with two ground joints, reduced pressure was applied, the liquid in the reaction flask was stirred at room temperature, and the receiver flask was cooled with liquid nitrogen.

Unless otherwise stated, thin layer chromatography (TLC) was performed on silica gel-coated aluminum foil (Merck, 60 F254), and the spots were detected with UV light (254 nm). For manual column chromatography, silica gel 60 M (Macherey-Nagel) was used as the stationary phase and slight pressure was applied. In the preparation procedures described below, the size of the column is given as diameter × length. Preparative HPLC was performed with UV detection at 254 nm using a Phenomenex Luna silica(2) column (particle size 5 µm, pore size 100 Å, column size 21.2 mm × 250 mm) at room temperature. The composition of solvent mixtures is given in volume ratios.

Unless otherwise stated, NMR spectra were calibrated using the signal of the solvent as an internal standard [$CDCl_3$: δ(^{1}H of $CHCl_3$)= 7.26 ppm, δ(^{13}C of $CDCl_3$) = 77.16 ppm; CD_2Cl_2: δ(^{1}H of $CHDCl_2$) = 5.32 ppm, δ(^{13}C of CD_2Cl_2) = 53.84 ppm. The given ratio of components in a mixture is a molar ratio and was determined by ^{1}H NMR spectroscopy.

ESI mass spectra were recorded using an Esquire 3000 ion trap mass spectrometer (Bruker Daltonik) equipped with a nano-ESI source. Accurate ESI mass measurements were acquired using a Q-IMS-TOF mass spectrometer Synapt G2Si (Waters, Manchester, UK) in the resolution mode, interfaced with a nano-ESI ion source. Nitrogen, provided by the nitrogen generator NGM 11, served both as the nebulizer gas and the dry gas for nano-ESI. Helium (Linde, 5.0) was used as buffer gas in the IMS entry cell. Nitrogen (Linde, 5.0) was used for ion mobility separation. Samples

were introduced by static nano-ESI using emitters pulled in-house from glass capillaries. The mono-isotopic masses of the compounds are reported.

Syntheses

$P_{R2}EP_{(R2)2}$-I 3. A 1.0 M solution of $^{n}Bu_4NF$ in THF (390 µL, 390 µmol) was added to a solution of $P_{R1}EP_{(R1)2}$-I **2**(Soetbeer et al., 2021) (101 mg, 108 µmol) in THF (5 mL), whereupon the color of the solution turned immediately from yellow to orange-brown. The solution was stirred at room temperature for 10 min and filtered through silica gel (3.0 cm × 3.0 cm, rinsing with THF). After removal of the solvent, a mixture (96 mg) of $P_{R2}EP_{(R2)2}$-I **3** and TIPS-X (X most probably being F) along with a small amount of $^{n}Bu_4NY$ (Y = F, OH) and THF was obtained as a pale orange solid. ^1H NMR (500 MHz, $CDCl_3$): δ = 7.51 (one half of an AA'XX' spin system, 2 H, $H_{benzene}$ *meta* to O), 7.48 (s, 1 H, $H_{benzene}$ *ortho* to I), 7.09 (s, 1 H, $H_{benzene}$ *meta* to I), 6.96 (other half of the AA'XX' spin system, 2 H, $H_{benzene}$ *ortho* to O), 4.77, 4.73, and 4.72 (3 d, ^4J = 2.4 Hz, 2 H, $C\underline{H}_2C{\equiv}CH$), 2.563, 2.561, and 2.541 (3 t, ^4J = 2.4 Hz, $CH_2C{\equiv}C\underline{H}$).

$P_{R3}EP_{(R3)2}$-I 5. This reaction was performed under argon. PEG azide **4**(Qi et al., 2016) (168 mg, 397 µmol) and the above described mixture (96 mg) containing $P_{R2}EP_{(R2)2}$-I **3** was dissolved in anhydrous toluene (10 mL). The yellow solution was degassed and $[Cu(phen)(PPh_3)_2]NO_3{\bullet}0.5\ CH_2Cl_2$ (6.4 mg, 7.7 µmol) was added. The yellow solution was stirred at 40 °C for 50 hours. The solution was cooled to room temperature. Metal scavenger QuadraPure® TU (78 mg) was added and the suspension was stirred at room temperature for 3 hours. The mixture was filtered and the solvent of the filtate was removed. The residual yellow oil was dissolved in CH_2Cl_2 (3 mL), metal scavenger QuadraPure® BzA (126 mg) was added, and the suspension was stirred at room temperature for another 2 days. The suspension was filtered. Removal of the solvents

from the filtrate gave a yellow oil (292 mg). Column chromatography (2.0 cm × 30 cm, CH_2Cl_2/EtOH 10:1) of this oil gave $P_{R3}EP_{(R3)2}$-I **5** (128 mg, 68% yield over two steps referring to $P_{R1}EP_{(R1)2}$-I **2**; R_f = 0.12) as a yellow oil. ^{1}H NMR (500 MHz, $CDCl_3$): δ = 7.88 and 7.85 (2 s, 2 H and 1 H respectively, $H_{triazole}$), 7.43 (s, 1 H, $H_{benzene}$ *ortho* to I), 7.42 (one half of an AA'XX' spin system, 2 H, $H_{benzene}$ *meta* to O), 7.06 (s, 1 H, $H_{benzene}$ *meta* to I), 6.95 (other half of the AA'XX' spin system, 2 H, $H_{benzene}$ *ortho* to O), 5.26 and 5.20 (2 s, 4 H and 2 H respectively, $C_{benzene}OCH_2$), 4.52, 4.51 and 4.49 (3 d, ^3J = 6.3 Hz, 2 H each, $N_{triazole}CH_2$), 3.67−3.45 (m, 84 H, $OC\underline{H}_2C\underline{H}_2O$), 3.41−3.29 (m, 12 H, $CH(C\underline{H}_2)_2$), 3.35, 3.33, and 3.31 (3 s, 6 H each, OCH_3), 2.48 (m, 3 H, $C\underline{H}(CH_2)_2$). ^{13}C NMR (126 MHz, $CDCl_3$): δ = 158.7, 153.9, and 151.9 ($C_{benzene}O$), 143.41, 143.38, and 143.21 ($\underline{C}_{triazole}CH_2$), 133.2 ($HC_{benzene}$-$\underline{C}_{benzene}H$ *meta* to O), 125.7 ($C_{triazole}H$), 125.0 ($C_{triazole}H$ and $C_{benzene}H$ *ortho* to I), 124.8 ($C_{triazole}H$), 117.0 ($C_{benzene}H$ *meta* to I), 115.7 and 115.1 ($\underline{C}_{benzene}C{\equiv}C\underline{C}_{benzene}$), 115.0 ($H\underline{C}_{benzene}$-$C_{benzene}H$ *ortho* to O), 94.9 ($C_{benzene}\underline{C}{\equiv}C$ *para* to O), 87.1 (C-I), 84.1 ($C_{benzene}C{\equiv}\underline{C}$ *para* to I), 72.02, 72.01, 72.00, 70.75, 70.74, 70.71, 70.69, 70.66, 70.62, 70.61, 70.58, 70.49, 70.47, and 70.42 ($O\underline{C}H_2\underline{C}H_2O$), 69.1 and 69.0 ($CH(\underline{C}H_2)_2$), 64.5, 64.3 and 62.1 ($C_{triazole}$-$\underline{C}H_2$), 59.13, 59.11, and 59.09 (OCH_3), 48.7 and 48.6 ($N_{triazole}CH_2$), 40.60 and 40.58 ($N_{triazole}CH_2\underline{C}H$). In addition to ^{1}H and ^{13}C NMR spectra, ^{13}C DEPT 135, ^{1}H,^{1}H-COSY, ^{1}H,^{13}C-HMQC, and ^{1}H,^{13}C-HMBC NMR spectra were recorded (see data depository). Accurate MS (ESI): *m/z* calcd for $[M + 2\ Na]^{2+}$ $C_{77}H_{126}N_9O_{27}INa_2^{2+}$, 890.8796; found, 890.8790.

Acetanilide 8. 2-Iodo-5-acetylaminophenol (**6**)(Teucher et al., 2020) (1.50 g, 5.41 mmol) and PEG-tosylate **7**(Teucher et al., 2020) (2.07 g, 6.49 mmol) were dissolved in acetonitrile (120 mL). Na_2CO_3 (1.72 g, 16.2 mmol) and KI (0.91 g, 5.4 mmol) were added to the solution. The suspension was stirred at 75 °C for 16 h. ^{1}H NMR spectroscopy revealed an incomplete conversion. The suspension was stirred at 90 °C

under reflux for 44 h. The suspension was cooled to room temperature. Water (70 mL) was added and the phases were mixed well. The two phases were separated. The aqueous phase was extracted with Et_2O (5 × 100 mL). The combined organic phases were dried over Na_2SO_4 and filtered. Removal of the solvents gave a pale-brown oil (2.56 g). Column chromatography of this oil (5.0 cm × 46 cm, CH_2Cl_2/EtOH, 15:1) gave an 87:13 mixture (1.09 g) of acetanilide **8** (R_f = 0.45) and 2-iodo-5-acetylaminophenol (**6**; R_f = 0.42) as a pale-yellow oil. This pale-yellow oil was dissolved in Et_2O (15 mL) and CH_2Cl_2 (15 mL) and was washed with 1.0 M aqueous Na_2CO_3 solution (3 × 15 mL). Removal of the solvent gave acetanilide **8** (880 mg, 38%) along with a small amount of CH_2Cl_2 and a trace of other unidentified compounds as a pale-yellow oil. ^{1}H NMR (500 MHz, $CDCl_3$): δ = 7.63 (d, ^3J = 8.4 Hz, 1 H, $H_{benzene}$ *meta* to O), 7.40 (br s, 1 H, NH), 7.34 (d, ^4J = 1.8 Hz, 1 H, $H_{benzene}$ *ortho* to O), 6.80 (dd, ^3J = 8.4 Hz, ^4J = 1.8 Hz, 1 H, $H_{benzene}$ *para* to O), 4.17, 3.90, 3.80, 3.68, and 3.57 (5 m, 2 H, 2 H, 2 H, 4 H, and 2 H respectively, OCH_2), 3.38 (s, 3 H, OCH_3), 2.16 (s, 3 H, O=CCH_3). ^{13}C NMR (126 MHz, $CDCl_3$): δ = 169.0 (C=O), 157.7 ($C_{benzene}$O), 140.0 ($C_{benzene}$N), 139.0 ($C_{benzene}$H *meta* to O), 114.0 ($C_{benzene}$H *para* to O), 104.74 ($C_{benzene}$H *ortho* to O), 79.4 ($C_{benzene}$I), 72.0, 71.2, 70.8, 70.5, 69.6, and 69.2 (OCH_2), 59.0 (OCH_3), 24.7 (O=$C\underline{C}H_3$). MS (ESI): *m/z* = 462.02 $[M + K]^+$, 446.09 $[M + Na]^+$, 424.12 $[M + H]^+$.

Aniline 9a. Acetanilide **8** (880 mg, 2.08 mmol) was dissolved in methanol (15.0 mL) and a solution of KOH (1.40 g, 20.8 mmol) in water (10.0 mL) was added. The solution was stirred at 85 °C for 60 h. During this time a colorless solid formed. After cooling to room temperature, the suspension was filtered through silica gel (0.7 cm × 3.0 cm, rinsing with CH_2Cl_2/EtOH 15:1). Removal of the solvents from the eluate gave an 87:13 mixture (531 mg) of aniline **9a** (61% yield) and aniline **9b** (9% yield) along with a small amount of CH_2Cl_2 and a trace of other unidentified compounds as a pale orange oil.

^1H NMR signals assigned to aniline **9a** (500 MHz, $CDCl_3$): δ = 7.44 (d, ^3J = 8.3 Hz, 1 H, $H_{benzene}$ *meta* to O), 6.24 (d, ^4J = 2.4 Hz, 1 H, $H_{benzene}$ *ortho* to O), 6.10 (dd, ^3J = 8.3 Hz, ^4J = 2.4 Hz, 1 H, $H_{benzene}$ *para* to O), 4.11, 3.90, 3.81, 3.68, and 3.56 (5 m, 2 H, 2 H, 2 H, 4 H, and 2 H respectively, OCH_2), 3.38 (s, 3 H, OCH_3). ^1H NMR signals assigned to aniline **9b** (500 MHz, $CDCl_3$): δ = 7.04 (t, ^3J = 8.1 Hz, 1 H, $H_{benzene}$ *meta* to O), 6.32 (m, 1 H, $H_{benzene}$ *para* to N), 6.29 (m, 1 H, $H_{benzene}$ *para* to O), 6.27 (m, 1 H, $H_{benzene}$ *ortho* to N and *ortho* to O), 4.09, 3.83, 3.73, 3.68, and 3.56 (5 m, 2 H, 2 H, 2H, 4 H, 2 H respectively, OCH_2), 3.38 (s, 3 H, OCH_3).

I-P_{R4}-NO• 12a. This reaction makes use of a procedure developed by H. Hintz.(Ritsch et al., 2019) It was performed in dried glassware under argon. Under cooling with an ice-water bath, thionyl chloride (202 µL, 2.78 mmol) was added to a yellow solution of 2,2,5,5-tetramethyl-3-pyrrolin-1-oxyl-3-carboxylic acid (**10**; 527 mg, 2.86 mmol) and 4-(*N*,*N*-dimethylamino)pyridine (880 mg, 7.20 mmol) in dry CH_2Cl_2 (15 mL), which made the color of the solution to change from yellow to dark orange. The ice-water bath was removed and the dark orange solution was stirred at room temperature for 35 min. Under cooling with an ice-water bath, a solution of the above described 87:13 mixture (527 mg) of aniline **9a** (1.26 mmol) and aniline **9b** (189 µmol) in dry CH_2Cl_2 (10 mL) was added. The cooling bath was removed, and the solution was stirred for 16 h. The solution was filtered through silica gel (0.7 cm × 3.0 cm, rinsing with CH_2Cl_2/EtOH, 15:1). The filtrate was washed with 1.0 M aqueous HCl (75 mL), 1.0 M aqueous $NaHCO_3$ solution (75 mL), and saturated aqueous NaCl solution (75 mL), dried over $MgSO_4$, and filtered. The solvents of the filtrate were removed. Column chromatography (2.0 cm × 30 cm, CH_2Cl_2/EtOH 21:1) of the residue (825 mg) gave a 86:14 mixture (674 mg) of I-P_{R4}-NO• **12a** (87% yield referring to aniline **9a**; R_f = 0.29) and H-P_{R4}-NO• **12b** (94% yield referring to aniline **9b**; R_f = 0.29) along with a small

amount of silicone grease and a trace of other unidentified compounds as a yellow oil. ^{1}H NMR signals assigned to I-P$_{R4}$-NO• **12a** (500 MHz, CD_2Cl_2): All signals are broad and featureless and integrals in the low field region come with a large error. δ = 7.72 (s, 1 H, H$_{benzene}$ *meta* to O), 7.35 (s, H$_{benzene}$ *ortho* to O), 6.88 (s, 1 H, H$_{benzene}$ *para* to O), 4.20, 3.89, 3.75, 3.64, 3.61, and 3.57 (6 m, 2 H each, OCH_2), 3.33 (s, 3 H, OCH_3). ^{1}H NMR signals assigned to H-P$_{R4}$-NO• **12b** (500 MHz, CD_2Cl_2): All signals are broad and featureless and integrals in the low field region come with a large error. δ = 7.26 (s, 1 H, H$_{benzene}$ *meta* to O), 7.04 (s, 1 H, *para* to O), 6.68 (d, 1 H, H$_{benzene}$ *para* to N), 4.13, 3.82, 3.68 (3 m, 2 H each). Other signals cannot be identified due to the overlap with the signals of I-P$_{R4}$-NO• **12a**. MS (ESI): *m/z* = 570.10 [**12a** + Na]$^+$, 565.16 [**12a** + NH_4]$^+$, 548.16 [**12a** + H]$^+$, 444.27 [**12b** + Na]$^+$, 439.34 [**12b** + NH_4]$^+$, 422.31 [**12b** + H]$^+$.

TIPS-EP$_{R4}$-NO• 13. This reaction was performed under argon. A solution of (triisopropylsilyl)acetylene (TIPS-acetylene, 173 µL, 767 µmol) and the above described 86:14 mixture (305 mg) of I-P$_{R4}$-NO• **12a** (493 µmol) and H-P$_{R4}$-NO• **12b** (81 µmol) in piperidine (280 µL, 2.84 mmol) and THF (12 mL) was degassed. $PdCl_2(PPh_3)_2$ (4.70 mg, 6.72 µmol) and CuI (2.87 mg, 14.6 µmol) were added. The yellow solution was stirred at room temperature for 5 days. H_2O (5 mL), 1.0 M aqueous HCl (5 mL) and CH_2Cl_2 (20 mL) were added and the organic and the aqueous phases were separated. The aqueous phase was extracted with CH_2Cl_2 (2 × 20 mL). The combined organic phases were dried over $MgSO_4$, filtered, and concentrated to ~15 mL. The concentrated organic phase was filtered through silica gel (0.7 cm × 3.0 cm, rinsing with CH_2Cl_2/EtOH, 15:1) and the filtrate was concentrated at reduced pressure. TIPS-EP$_{R4}$-NO• **13** was isolated using preparative HPLC employing an isocratic elution at a flow rate of 12 mL/min. The mobile phase consisted of CH_2Cl_2/EtOH 97.0:3.0. The eluate between 12.5 and 14.0 min was collected. Removal of the solvents gave TIPS-

EP_{R4}-NO• **13** (119 mg, 36%) as a yellow-orange oil. ^1H NMR (500 MHz, CD_2Cl_2): All signals are broad and featureless and integrals in the low field region come with a large error. δ = 7.43 (s, 1 H, $H_{benzene}$ *meta* to O), 7.26 (s, 1 H, $H_{benzene}$ *para* to O), 7.04 (s, 1 H, $H_{benzene}$ *ortho* to O), 4.19, 3.85, 3.71, 3.64, and 3.53 (5 m, 2 H, 2 H, 2 H, 4 H, and 2 H respectively, OCH_2), 3.34 (s, 3 H, OCH_3), 1.16 (s, 21 H, $Si(C\underline{H}(C\underline{H}_3)_2)_3$). ^{13}C NMR (126 MHz, CD_2Cl_2): δ = 159.9 ($C_{benzene}O$), 133.8 ($C_{benzene}H$ *meta* to O), 119.7 ($C_{benzene}H$ *para* to O), 109.4 ($\underline{C}_{benzene}$-C≡C), 102.6 ($\underline{C}$≡C-TIPS), 94.3 (C≡$\underline{C}$-TIPS), 71.9, 70.8, 70.5, 70.3, 69.5, and 68.6 (OCH_2), 58.6 (OCH_3), 18.4 ($CH(\underline{C}H_3)_2$), 11.3 ($\underline{C}H(CH_3)_2$). MS (ESI): *m/z* = 640.32 $[M + K]^+$, 624.46 $[M + Na]^+$, 619.46 $[M + NH_4]^+$, 602.44 $[M + H]^+$.

H-EP_{R4}-NO• 14. A 1.0 M solution of $^nBu_4NF$ in THF (0.5 mL, 0.5 mmol) was added to a solution of TIPS-EP_{R4}-NO• **13** (241 mg, 400 µmol) in THF (5 mL). The orange-brown solution was stirred at room temperature for 10 min. The solution was filtered through silica gel (3.0 cm × 3.0 cm, rinsing with CH_2Cl_2/EtOH, 20:1). Removal of the solvents from the eluate gave a brown oil (209 mg). Column chromatography of this oil (2.0 cm × 33 cm, CH_2Cl_2/Et_2O, 20:1) gave a mixture (R_f = 0.27; 87 mg) of TIPS-EP_{R4}-NO• **13** and H-EP_{R4}-NO• **14** as a yellow-brown oil. This oil was dissolved in THF (6 ml). A 1.0 M solution of ^nBuNF in THF (0.5 mL, 0.5 mmol) was added and the red-brown solution was stirred at room temperature for 18 hours. The solution was filtered through silica gel (1.5 cm × 5.0 cm, rinsing with CH_2Cl_2/EtOH, 5:1). Removal of the solvents from the eluate gave a brown oil (90 mg). H-EP_{R4}-NO• **14** was isolated using preparative HPLC employing an isocratic elution at a flow rate of 12 mL/min. The mobile phase consisted of CH_2Cl_2/EtOH 97.0:3.0. The eluate between 15.6 and 16.2 min was collected. Removal of the solvents gave H-EP_{R4}-NO• **14** (38 mg, 21%) as a yellow oil. ^1H NMR (500 MHz, $CDCl_3$): All signals are broad and featureless and integrals in the low field region come with a large error. δ = 7.44 (s, 1 H, $H_{benzene}$ *meta* to O), 7.26 (s, 1 H, *para*

to O), 6.97 (s, 1 H, *ortho* to O), 4.26, 3.92, 3.80, 3.69, 3.68, and 3.57 (6 m, 2 H each, OCH_2), 3.39 (s, 3 H, OCH_3), 3.22 (s, 1 H, HC≡C).

Model compound 1, $P_{R3}EP_{(R3)2}EP_{R4}$-NO•. This reaction was performed under argon. A solution of $P_{R3}EP_{(R3)2}$-I **5** (37.9 mg, 21.8 μmol) and H-EP_{R4}-NO• **14** (12.7 mg, 28.5 μmol) in piperidine (1.0 mL, 10 mmol) and THF (5 mL) was degassed. $Pd(PPh_3)_4$ (1.61 mg, 1.39 μmol), $Pd_2(dba)_3$ (1.38 mg, 1.51 μmol), and CuI (0.99 mg, 5.20 μmol) were added. The yellow solution was stirred at room temperature for 20 hours and then all volatiles were removed via short path distillation. The residue was dissolved in CH_2Cl_2 (dry, degassed; 3 mL), metal scavenger QuadraPure® TU (27 mg) was added and the suspension was stirred for 21.5 hours. The suspension was filtered and the solvent was removed. $P_{R3}EP_{(R3)2}EP_{R4}$-NO• **1** was isolated using preparative HPLC (Figure S1) with a linear gradient elution at a flow rate of 12 mL/min. The mobile phase consisted of CH_2Cl_2 and EtOH with the following percentages of EtOH: 0−10 min, 5%; 10−20 min, 5%−10%; 20−30 min, 10%−20%; 30−50 min, 20%. The eluate between 12.9 and 15.8 min was collected. Removal of the solvents gave alkyne dimer •ON-$P_{R4}EEP_{R4}$-NO• **15** (Glaser coupling product; 5.2 mg, 41%) as a yellow oil. The eluate between 23.7 and 25.7 min was collected. Removal of the solvents gave $P_{R3}EP_{(R3)2}EP_{R4}$-NO• **1** (20.3 mg, 45%) as a yellow oil. The purity of model compound **1** was checked by analytical HPLC (Figure S2): The analytical HPLC was performed with UV detection at 254 nm using a Phenomenex Luna silica(2) column (particle size 5 μm, pore size 100 Å, column size 4.6 mm × 250 mm) at room temperature with a linear gradient elution at a flow rate of 1.0 mL/min. The mobile phase consisted of CH_2Cl_2 and EtOH with the following percentages of EtOH: 0−10 min, 5%; 10−20 min, 5%−10%; 20−30 min, 10%−20%; 30−50 min, 20%.

Analytical data of alkyne dimer •ON-$P_{R4}EEP_{R4}$-NO• **15**: ^{1}H NMR (500 MHz, $CDCl_3$): All signals are broad and featureless and integrals come with a large error. 7.47 (s, 2 H, $H_{benzene}$ *meta* to R^4), 7.05 (s, 4 H, $H_{benzene}$ *ortho* and *para* to R^4), 4.28, 3.94, 3.85, 3.73, 3.69, and 3.58 (6 s, 4 H each, OCH_2), 3.38 (s, 6 H, OCH_3). MS (ESI): *m*/*z* = 887.3 [M + H]$^+$.

Analytical data of model compound $P_{R3}EP_{(R3)2}EP_{R4}$-NO• **1**: ^{1}H NMR (500 MHz, $CDCl_3$): All signals are broad and featureless and integrals in the low field region come with a large error. δ = 7.91 and 7.88 (2 s, 2 H and 1 H respectively, $H_{triazole}$), 7.46 (one half of an AA'XX' spin system, 2 H, $H_{benzene}$ *meta* to R^3), 7.42 (s, 1 H, $H_{benzene}$ *meta* to R^4), 6.99 (other half of the AA'XX' spin system, 2 H, $H_{benzene}$ *ortho* to R^3), 7.14 (s, 2 H, $H_{benzene}$ *ortho* to R^3), 5.31, 5.29, and 5.23 (3 s, 2 H each, $C_{benzene}OCH_2$), 4.54 and 4.52 (2 d, ^3J = 6.1 Hz, 2 H each, $N_{triazole}CH_2$), 4.44 (s, 2 H, $N_{triazole}CH_2$), 4.26, 3.88, and 3.74 (3 s, 2 H each, OCH_2 of R^4), 3.73−3.45 (m, 90 H, OCH_2 of R^3 and R^4), 3.44−3.25 (m, 12 H, $CH(C\underline{H}_2)_2$), 3.36 and 3.35 (2 s, 12 H each, OCH_3 of R^3 and R^4), 2.51 (m, 2 H, $C\underline{H}(CH_2)_2$), 2.30 (s, 1 H, $C\underline{H}(CH_2)_2$). ^{13}C NMR (126 MHz, $CDCl_3$): δ = 158.2, 153.2, and 153.0 ($C_{benzene}O$), 144.0, 143.2, and 142.8 ($\underline{C}_{triazole}CH_2$), 132.8 ($HC_{benzene}$-$\underline{C}_{benzene}H$ *meta* to R^3), 124.6, 124.4, and 123.9 ($C_{triazole}H$), 118.3 and 117.9 ($C_{\textit{benzene}}H$ *ortho* to R^3), 115.4 and 114.8 ($\underline{C}_{benzene}C{\equiv}C\underline{C}_{benzene}$), 114.6 ($H\underline{C}_{benzene}$-$C_{benzene}H$ *ortho* to O), 95.0 ($C_{benzene}\underline{C}{\equiv}C$ *para* to R^3), 91.3, 88.9, and 84.1 ($C{\equiv}\underline{C}$ *ortho* to R^3), 71.58, 71.57, 70.62, 70.36, 70.30, 70.28, 70.23, 70.18, 70.14, 70.11, 70.05, 69.99, and 69.12 (OCH_2CH_2O), 68.65, 68.62, and 68.59 ($CH(\underline{C}H_2)_2$), 64.5, 63.7 and 61.7 ($C_{triazole}\underline{C}H_2$), 58.80, 58.68, and 58.66 (OCH_3), 48.32, 48.25 and 48.23 ($N_{triazole}CH_2$), 40.24, 40.19, and 40.16 ($N_{triazole}CH_2\underline{C}H$). Accurate MS (ESI): *m/z* calcd for [M + 2 Na]$^{2+}$ $C_{101}H_{158}N_{11}O_{33}Na_2^{2+}$, 1049.5404; found, 1049.5403.

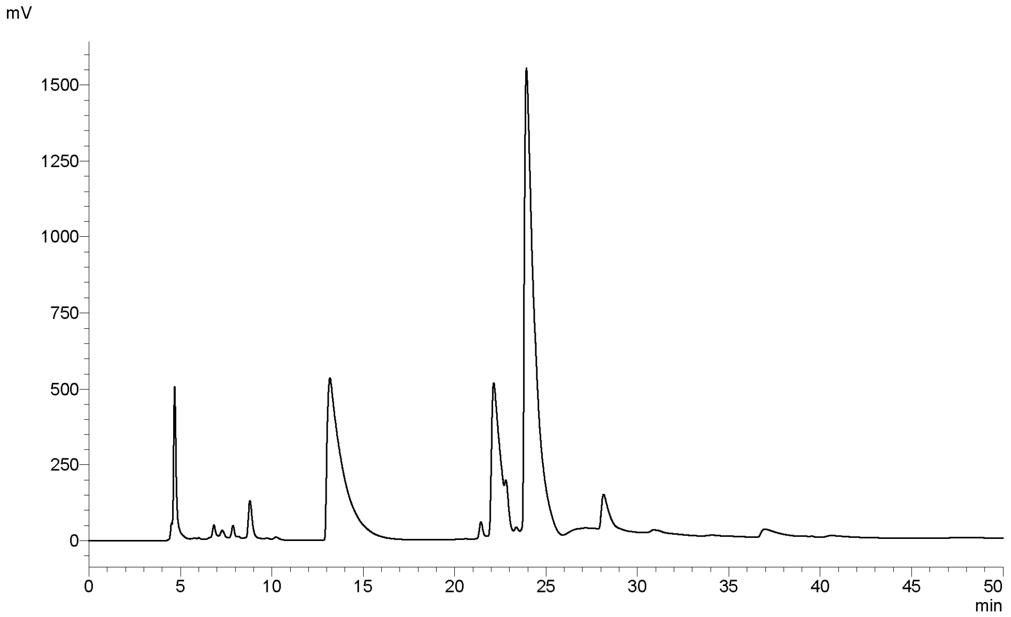

Figure S1. Chromatogram of the preparative HPLC of the product mixture obtained in the synthesis of model compound **1**. The alkyne dimer •ON-P_{R4}EEP_{R4}-NO• (**15**) was eluted between 12.9 and 15.8 min, the model compound P_{R3}E$P_{(R3)2}$EP_{R4}-NO• **1** was eluted between 23.7 and 25.7 min.

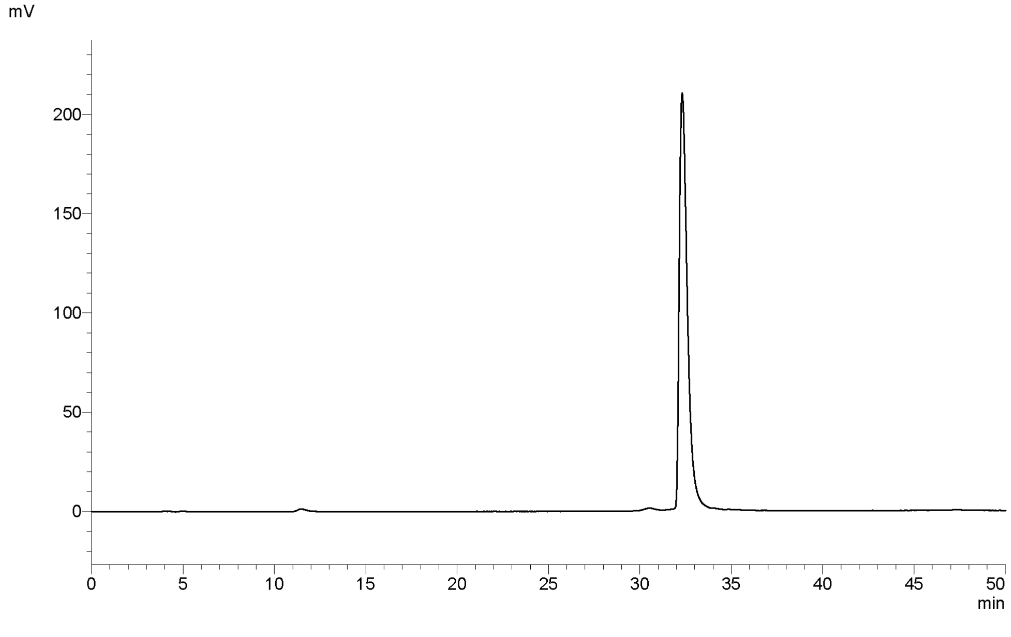

Figure S2. Analytical HPLC chromatogram of the fraction obtained through preparative HPLC consisting of model compound **1**.

References

Kukula, H., Veit, S., and Godt, A.: Synthesis of monodisperse oligo(*para*-phenyleneethynylene)s using orthogonal protecting groups with different polarity for terminal acetylene units, Eur. J. Org. Chem., 277–286, https://doi.org/10.1002/(SICI)1099-0690(199901)1999:1<277:AID-EJOC277>3.0.CO;2-R, 1999.

Qi, M., Hülsmann, M., and Godt, A.: Spacers for geometrically well-defined water-soluble molecular rulers and their application, J. Org. Chem., 81, 2549–2571, https://doi.org/10.1021/acs.joc.6b00125, 2016.

Ritsch, I., Hintz, H., Jeschke, G., Godt, A., and Yulikov, M.: Improving the accuracy of Cu(II)-nitroxide RIDME in the presence of orientation correlation in water-soluble Cu(II)-nitroxide rulers, Phys. Chem. Chem. Phys., 21, 9810–9830, https://doi.org/10.1039/c8cp06573j, 2019.

Soetbeer, J., Millen, M., Zouboulis, K., Hülsmann, M., Godt, A., Polyhach, Y., and Jeschke, G.: Dynamical decoupling in water-glycerol glasses: a comparison of nitroxides, trityl radicals and gadolinium complexes, Phys. Chem. Chem. Phys., 23, 5352–5369, https://doi.org/10.1039/D1CP00055A, 2021.

Teucher, M., Qi, M., Cati, N., Hintz, H., Godt, A., and Bordignon, E.: Strategies to identify and suppress crosstalk signals in double electron–electron resonance (DEER) experiments with gadolinium^{III} and nitroxide spin-labeled compounds, Magn. Reson., 1, 285–299, https://doi.org/10.5194/mr-1-285-2020, 2020.
