# Supplementary material for: ih-RIDME: a pulse EPR experiment to probe the heterogeneous nuclear environment
Source: Magn Reson (Gott). 2025 Mar 10;6(1):93–112. doi: 10.5194/mr-6-93-2025 (PMC12247092; doi:10.5194/mr-6-93-2025)
Supplement: The supplement related to this article is available online at https://doi.org/10.5194/mr-6-93-2025-supplement. [file mr-6-93-2025-supplement.zip › mr-2024-19_supplement_part_2.pdf]

**Contents**


**S2 Solution of spectral diffusion equation and an approximate ih-RIDME kernel** **16**

**S3 Mean value and standard deviation from fitting the noised data** **17**

**S4 Tikhonov regularization** **18**

**S5 EPR data on the model compound 1** **19**
S5.1 Echo-detected field sweep EPR spectrum . . . . . 19
S5.2 Hahn echo decay . . . . . 19
S5.3 ih-RIDME data . . . . . 20
S5.4 ih-RIDME fitting . . . . . 21

**S6 Molecular modeling** **23**
S6.1 General description . . . . . 23
S6.2 Comparison of dihedral potentials . . . . . 23
S6.3 Convergence of the dataset . . . . . 24
S6.4 Comparison of the experimental and calculated $p(\sigma)$ . . . . . 25
S6.5 Ensemble representation with blocking radius . . . . . 26

**S7 ih-RIDME sequences** **28**
S7.1 Remote detection in 3p-RIDME . . . . . 28
S7.2 ESEEM averaging in 4p-RIDME . . . . . 28
S7.3 Comparison of the longitudinal factors . . . . . 29
S7.4 Extraction of transverse factors $F(t)$ . . . . . 29

## S2 Solution of spectral diffusion equation and an approximate ih-RIDME kernel

The evolution equation of the magnetization spectrum, proposed in Ref. Kuzin et al. (2022), is

$$\frac{\partial \mu(\omega_{\text{hf}},T)}{\partial T} = D\left(\rho(\omega_{\text{hf}})\mu''_{\omega\omega}(\omega_{\text{hf}},T) - \rho''(\omega_{\text{hf}})\mu(\omega_{\text{hf}},T)\right) \tag{S1}$$

In the absence of the analytical solution of Eq. (S1), we represent $\mu_t(\omega_{\text{hf}})$ in a basis of Hermitian functions

$$\mu_t(\omega_{\text{hf}},T_{\text{mix}}) = \frac{1}{\sqrt{2\pi}\sigma}\sum_{k=0}^{K} c_t^{(k)}(T_{\text{mix}})He_k\left(\frac{\omega_{\text{hf}}}{\sigma}\right)\exp\left(-\frac{\omega_{\text{hf}}^2}{2\sigma^2}\right) \tag{S2}$$

which allows to express $R(t)$ in matrix form(Kuzin et al., 2022)

$$R(t;T_{\text{mix}}) = \boldsymbol{c}_0{}^T\exp(-i\hat{R}\sigma t)\exp\left(\frac{D}{\sigma^3}\hat{\Gamma}T_{\text{mix}}\right)\exp(i\hat{R}\sigma t)\boldsymbol{c}_0 \tag{S3}$$

where $\boldsymbol{c}_0$ is a $(K+1)$-element column $(1,0,0,\ldots,0)^T$ and the matrices $\hat{R}$ and $\hat{\Gamma}$ are defined as

$$\left(\hat{R}\right)_{n,k} = \begin{cases} -1, \text{ if } n = k+1 \\ k, \text{ if } n = k-1 \\ 0 \text{ otherwise} \end{cases} \tag{S4}$$

$$\left(\hat{\Gamma}\right)_{n,k} = \begin{cases} 0, \text{ if } n=0 \text{ or } k=0 \text{ or } n+k \text{ is odd} \\ (-1)^k\dfrac{nk(n+k-3)!!}{n!\sqrt{4\pi}}\left(-\dfrac{1}{2}\right)^{\frac{n+k}{2}-1} \quad \text{otherwise.} \end{cases} \tag{S5}$$

$R(t;T_{\text{mix}})$ can be also written as a series

$$R(t;T_{\text{mix}}) = \exp\left(-\frac{\sigma^2t^2}{2}\right)\sum_{k=0}^{K} c_t^{(k)}(T_{\text{mix}})(-i\sigma t)^k \tag{S6}$$

where the vector $\boldsymbol{c}_t(T_{\text{mix}}) = \exp\left(\frac{D}{\sigma^3}\hat{\Gamma}T_{\text{mix}}\right)\exp(i\hat{R}\sigma t)\boldsymbol{c}_0$.

These forms are useful for mathematical justification of ih-RIDME traces congruence and for the introduction of the parameter $\kappa = D/\sigma^3$. However, these representations of $R(t;T_{\text{mix}})$ maybe less intuitive. Here, we decided to simplify this result by Gaussian fitting the ih-RIDME traces $R(t;T_{\text{mix}})$ calculated via the diffusion equation. We presented in Eq. (S3) in the main text that the longitudinal dynamics is parametrized by the unitless parameters $\varepsilon = DT_{\text{mix}}/\sigma^3$ and $\sigma t$. Thus, we calculated a family of ih-RIDME traces as functions of $\sigma t$ and parametrized by $\varepsilon$ followed by fitting them with $\exp(-\alpha(\varepsilon)\sigma^2t^2)$. The results are shown in Figure S3.

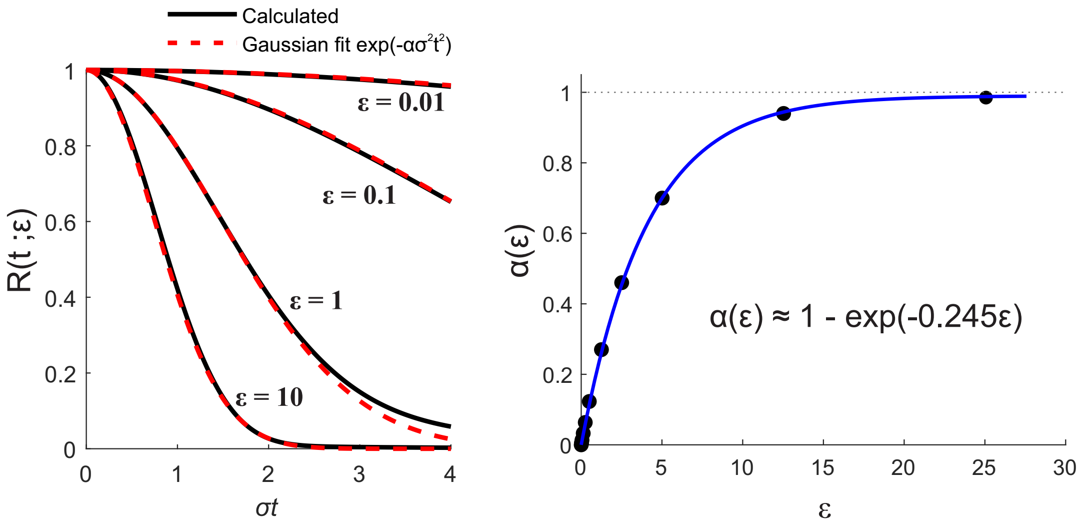


**Figure S3.** Left: examples of fitting of calculated time-domain RIDME traces by Gaussian function is a wide range of mixing times. Right: build-up of the extracted Gaussian curvature and its fitting.

## S3 Mean value and standard deviation from fitting the noised data

High noise level in ih-RIDME data leads to uncertainties in the fitted distribution $p(\sigma)$ (see Figure 5 in the main text). We show in Figure S4 that the mean value and the standard deviation are reproduced reliably from the fits. The data points were calculated using the distributions in Figure 5. The true values are mean $= 0.5$ MHz and std $= 0.18$ MHz.

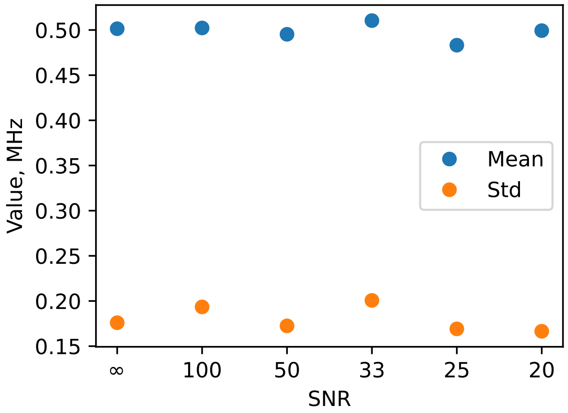


**Figure S4.** Mean values (blue dots) and standard deviations (orange dots) of fitted distributions $p(\sigma)$ from artificially noised data. The x-axis corresponds to the SNR of undivided data. The shapes of the distributions are shown in Figure 5 (main text).

## S4 Tikhonov regularization

Tikhonov regularization approach modifies the target minimization functional with a term $||\mathbf{L}_n p||$ where $\mathbf{L}_n$ is a matrix of a discrete $n^{\text{th}}$ derivative of the vector $p$. Traditionally, in PDS $n$ equals 2. The general minimization problem in ih-RIDME with the smoothness penalty is then

$$p(\sigma) = \arg\min_{p \geqslant 0} \left\{ \sum_{i=1}^{M-1} w_i \left\| \frac{V(t;T_{\text{mix},i})}{V(t;T_{\text{mix,ref}})} - \frac{\int K(\sigma t;T_{\text{mix},i}) p(\sigma) \mathrm{d}\sigma}{\int K(\sigma t;T_{\text{mix,ref}}) p(\sigma) \mathrm{d}\sigma} \right\| + \alpha^2 ||\mathbf{L}_2 p|| \right\}. \quad \text{(S7)}$$

The regularization parameter $\alpha^2$ tunes the weight of the smoothness penalty. In the top plot of Figure S5, we show fitted distributions of the noised dataset (SNR 20, see Section 4.3.2 in the main text) with different values of the regularization parameter.

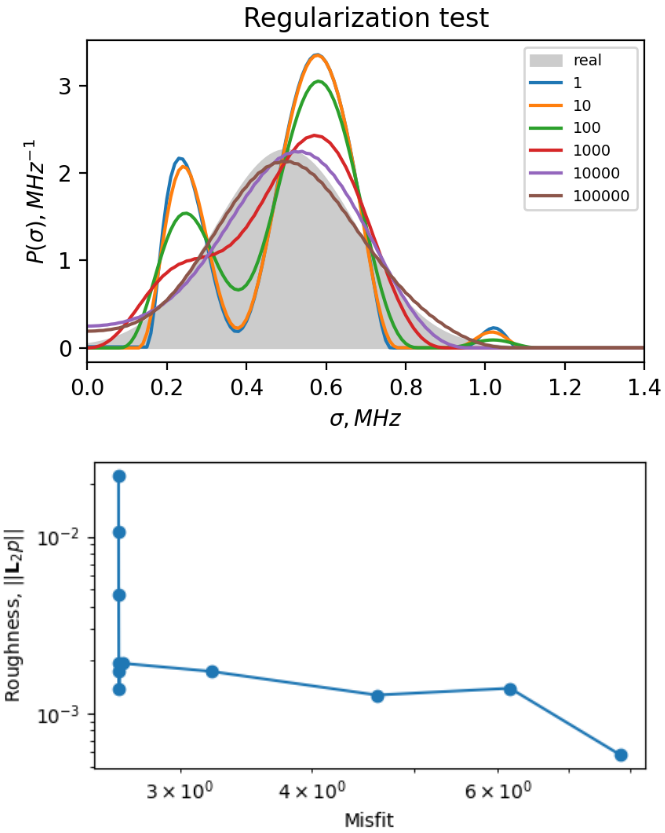


**Figure S5.** Top: fitted distributions $p(\sigma)$ for a noised dataset with different regularization parameter. Numbers in the legend correspond to the value of $\alpha^2$. Bottom: the corresponding L-curve.

The selection of the regularization parameter can be made based on a traditional L-curve criterion. The L-curve for the case under consideration is presented in Figure S5 (bottom). It features a distinct L-shape. The value in the corner of the L-curve corresponds to the brown distribution in the top panel.

## S5 EPR data on the model compound 1

### S5.1 Echo-detected field sweep EPR spectrum

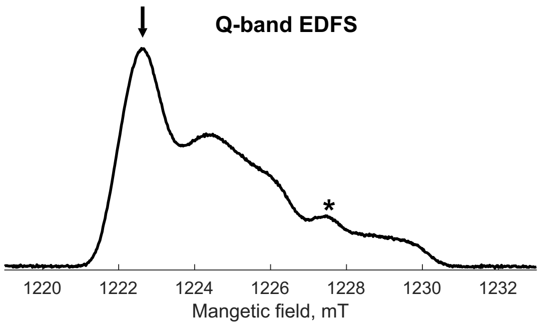


**Figure S6.** Q-band ($\nu_{mw} = 34.5$ GHz) echo-detected field sweep EPR spectrum of model compound **1** in a perdeuterated water-glycerol solvent. The arrow indicates the field position for the pulse EPR measurements. The pulses were set to $t_{\pi/2} = 12$ ns and $t_{\pi} = 24$ ns. The asterisk marks the radical defect in the quartz capillary.

### S5.2 Hahn echo decay

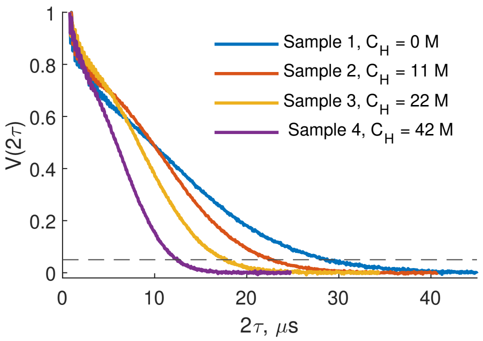


**Figure S7.** Normalized Hahn echo decay traces of the model compound **1** in partially deuterated water/glycerol. See Table 1 in the main text for details on the isotope composition. The measurements were done at Q band at 50 K. Dash line displays 5% of the initial intensity.

| Sample | $C_{\mathrm{H}}$(solvent) | $t_{0.05}$, $\mu s$ |
|---|---|---|
| **1** | 0 | 28.5 |
| **2** | 11.4 | 22.6 |
| **3** | 22.1 | 17.6 |
| **4** | 41.7 | 12.4 |

**Table S1.** Times of the Hahn echo decay to 5% of the initial amplitude of model compound **1** in water/glycerol solutions with different protonation degrees.

### S5.3 ih-RIDME data

The normalized raw data as well as the reference-divided traces are presented in Figure S8.

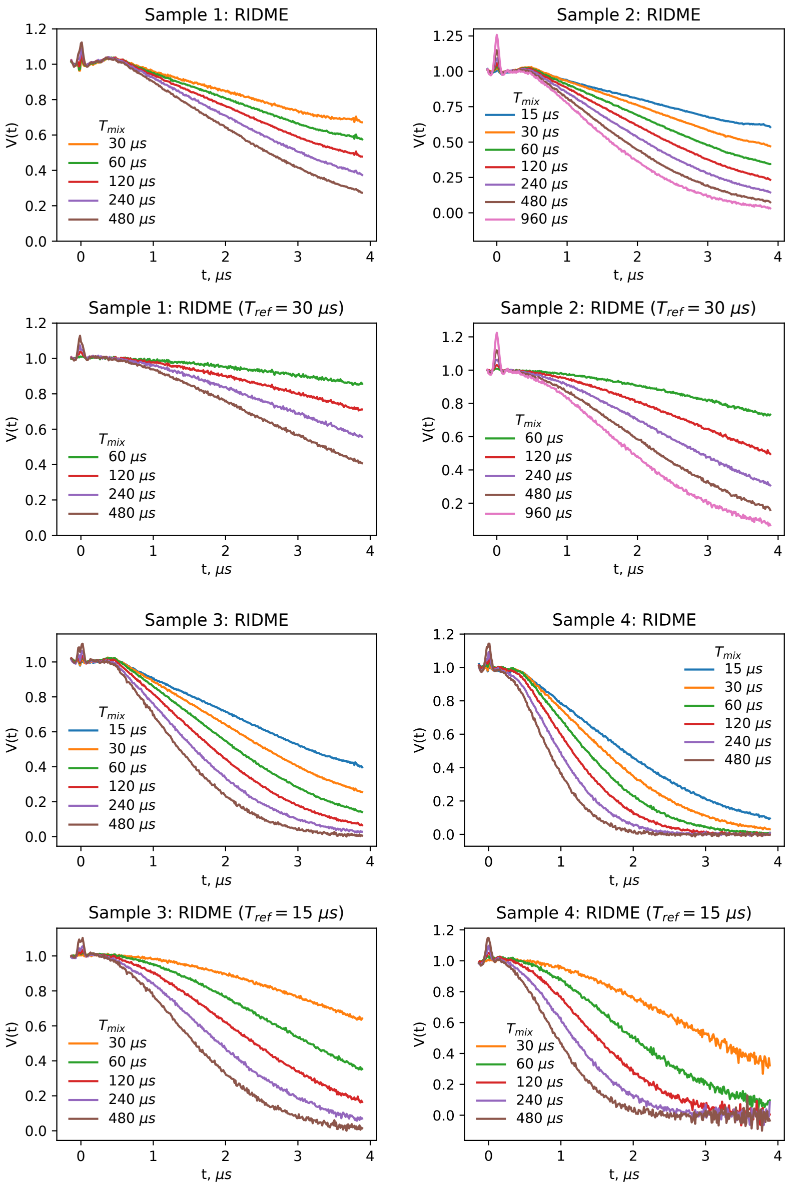


**Figure S8.** Normalized ih-RIDME traces and reference-divided ih-RIDME traces for four solutions of model compound **1**. See Table 1 in the main text for solvent composition.

### S5.4 ih-RIDME fitting

The fitting of reference-divided ih-RIDME traces was done with an approximate kernel

$$K(\sigma t; T_{\text{mix}}) = \exp(-(\alpha(T_{\text{mix}}) + \beta)\sigma^2 t^2) \tag{S8}$$

with

$$\alpha(T_{\text{mix}}) = 1 - \exp\left(-0.245\frac{D}{\sigma^3}T_{\text{mix}}\right). \tag{S9}$$

The visual comparison of reference-divided experimental and fitted data is given in Figure S9.

We analyzed the factor residuals of the undivided experimental data

$$G(t; T_{\text{mix}}) = \frac{V_{\text{exp}}(t; T_{\text{mix}})}{V_{\text{fit}}(t; T_{\text{mix}})} \tag{S10}$$

where $V_{\text{exp}}(t; T_{\text{mix}})$ are the experimental traces and $V_{\text{fit}}(t; T_{\text{mix}})$ are the traces computed for the optimized proton density distribution $p_{\text{opt}}(\sigma)$

$$V_{\text{fit}}(t; T_{\text{mix}}) = \int_{\sigma_{\text{min}}}^{\sigma_{\text{max}}} K(\sigma t; T_{\text{mix}}) p_{\text{opt}}(\sigma) \mathrm{d}\sigma\,. \tag{S11}$$

We found that the residuals are almost independent of mixing time and have characteristic roof-shaped features at $t \approx d_1$. The position of these features in the RIDME time axis slightly deviates from $d_1$ due to the used $^2$H-ESEEM averaging protocol(Keller et al., 2016) that increments the first interpulse delay from $d_1$ to $d_1 + 0.256\ \mu s$. A similar feature was observed in copper-nitroxide biradicals(Ritsch et al., 2019), however, it was not assigned to the presence of copper.

In general, the features found are not critical for the processing of ih-RIDME data. Due to $T_{\text{mix}}$-independence, the factor $G(t)$ is removed from the reference-divided dataset and does not interfere with the approximation in Eq. (S8). This is, however, an obstacle towards the fitting of unreferenced data, therefore, this effect should be investigated further.

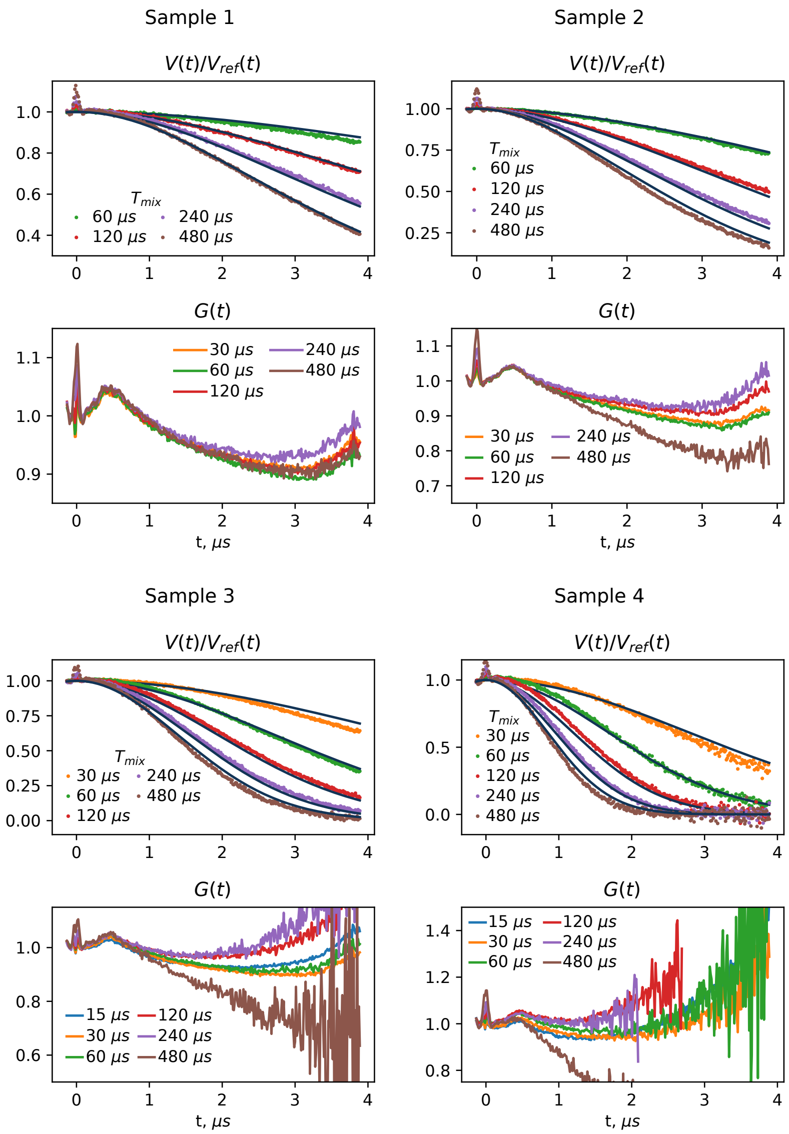


**Figure S9.** ih-RIDME data fits (black lines) and the residual factor $G(t)$ for a model compound **1** in different solvents. See Table 1 in the main text for details.

## S6 Molecular modeling

### S6.1 General description

The molecule was constructed from blocks with variable dihedral angles by a home-written MATLAB script. The blocks, which are shown in blue in Figure S10, were independently created in Avogadro software (Hanwell et al., 2012), optimized with MMFF94s force field (Halgren, 1999), and further merged. The O-C-C-O dihedral angles $\alpha$, $\beta$, and $\gamma$ were chosen from optimized potentials for liquid simulations (further denoted as OPLS) or modified OPLS profiles, which were taken from Ref. Hoffmann et al. (2023) and fitted by the sum of Gaussian distributions. The dihedral angles $\delta$ (C-C-O-C), $\varepsilon$ (H-C-C-H), $\zeta$ (C-N-C-C) and $\kappa$ (O-C-C-C) were chosen from the profile for methyl groups in ethane (Esquivel et al. (2011)) and fitted by the sum of Gaussian distributions. The dihedral angle probabilities are shown in Figure S11. The dihedral angle $\eta$ ($C_{23}-N-C_1-C_6$), the angle $\theta$ ($C_2-C_3-O-C$), the angle $\iota$ ($C_{10}-C_{11}-O-C$ and $C_{13}-C_{14}-O-C$) and the angle $\lambda$ ($C_{21}-C_{20}-O-C$) were canonical angles (0° and 180°). The $\mu$ ($C_5-C_4-C_9-C_{14}$) and $\nu$ ($C_{13}-C_{12}-C_{17}-C_{22}$) angles were canonical angles (0°). In a separate try, the low-amplitude libration near the canonical values for angles $\eta$, $\theta$, $\iota$, $\mu$ and $\nu$ was introduced. The distribution of libration amplitudes was taken Gaussian:

$$U(\alpha) = \frac{1}{\sqrt{2\pi}|\Delta\alpha|}\exp\left(-\frac{(\alpha-\alpha_{\text{canonic}})^2}{2|\Delta\alpha|^2}\right) \tag{S12}$$

with $\Delta\alpha = 7°$ for angles $\eta$, $\theta$, and $\iota$, and 5° for $\mu$ and $\nu$ angles. The results obtained with this modification are further referred to as "with libration".

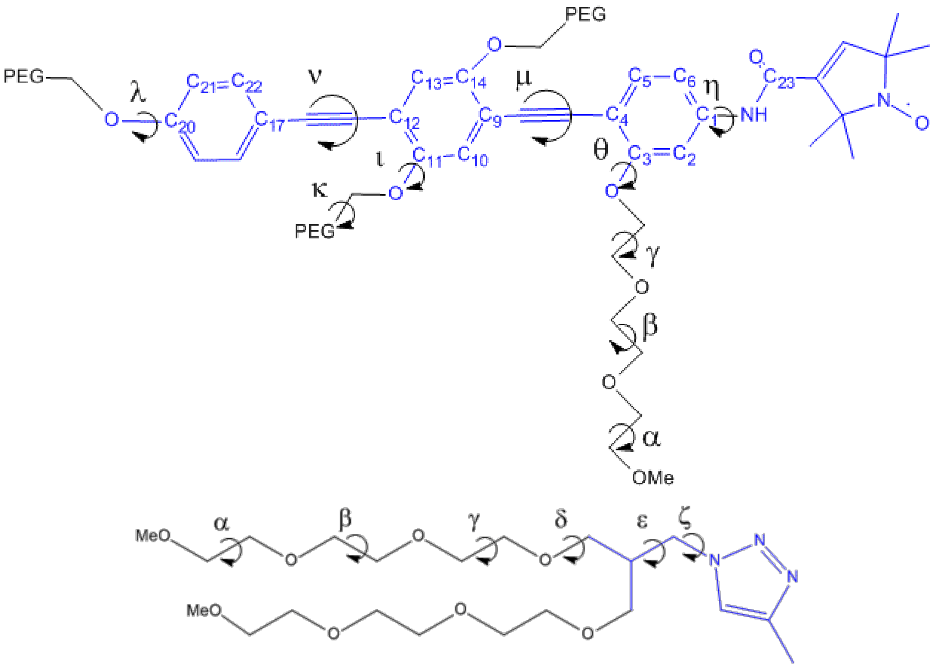


**Figure S10.** The molecular structure of model compound **1** and definition of variable dihedral angles.

### S6.2 Comparison of dihedral potentials

The ensembles were generated with four combinations: unmodified or modified OPLS dihedral potentials and without or with angle libration. We found that the obtained ensembles have rather similar characteristics. As an example, we show in

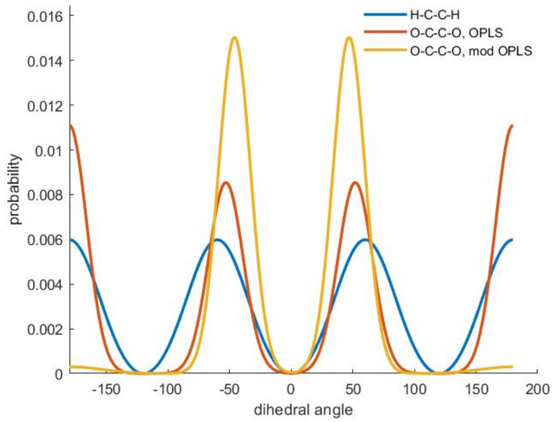


**Figure S11.** Dihedral angle probabilities for H-C-C-H in ethane (blue line) and O-C-C-O in OPLS (orange line) and modified OPLS ("mod OPLS", yellow line) potentials.

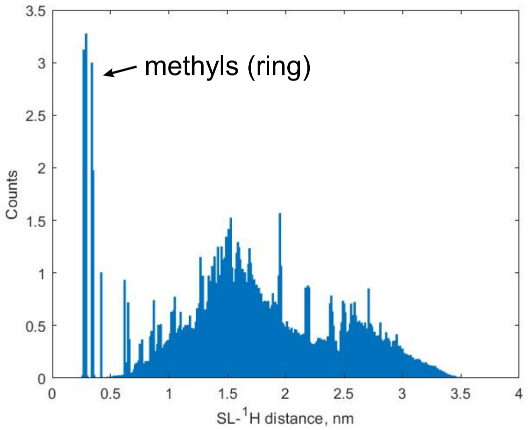


**Figure S12.** Spin-label-$^1$H distance distribution function for dataset constructed with OPLS profile and canonic $\eta$, $\theta$, $\iota$, $\lambda$, $\mu$ and $\nu$ angles.

Figure S13 the correlation plot for the maximal distance between the electron spin and the farthermost proton in a given conformer and the gyration radius of the same conformer. The first descriptor estimates the size of a molecule along the longest dimension. The second descriptor reflects the compactness of a molecule. Besides the slight correlation of these two values in the conformational ensemble, we observe that the four settings of the ensemble generation substantially overlap with each other. Therefore, we cannot give a preference to any of the models from the analysis of the ensemble.

### S6.3 Convergence of the dataset

We worked with a conformational ensemble of 1500 conformers. We tested that this number is sufficient by computing the distribution $p_n(\sigma)$ for subsets of variable sizes $n < 1500$ and comparing them with the $p(\sigma) = p_{1500}(\sigma)$ computed for the full set. As a result, we obtained a convergence plot in Figure S14. This curve has a steep feature at the beginning and reaches a shallow plateau after approximately 500 conformers. Further increase of the subset size has a weak impact on the convergence rate. Nonetheless, 1500 conformers of model compound **1** was still a computationally feasible size for a regular computer.

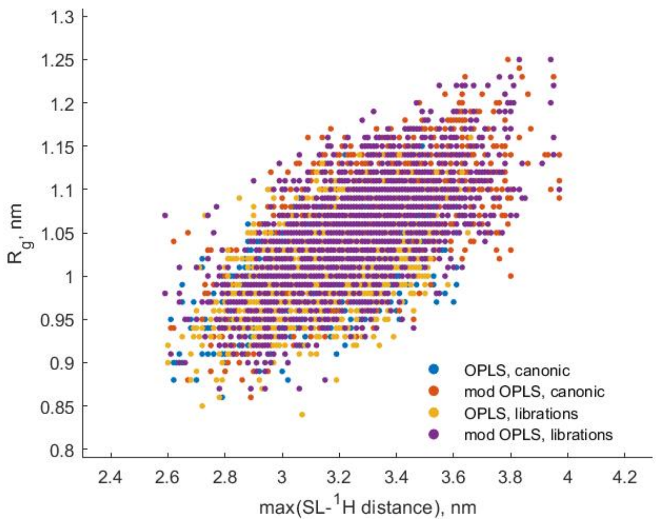


**Figure S13.** Correlation of the distance from the unpaired electron to the farthermost proton of a conformer (max(SL-$^1$H distance)) and the molecule's gyration radius ($R_g$) sampled over the generated conformational ensemble assuming OPLS or modified OPLS model with and without librations.

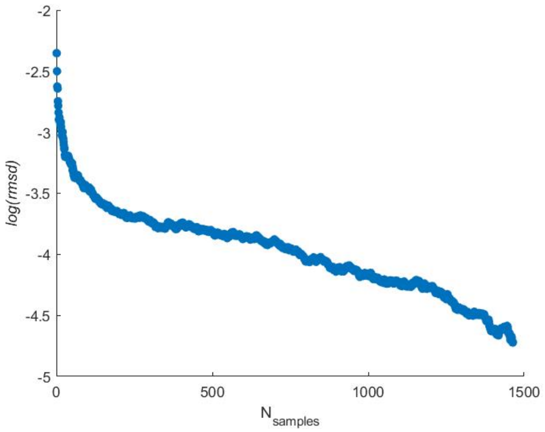


**Figure S14.** The rmsd between local proton densities averaged over the dataset with 1500 conformers, and for various numbers of conformers. Results are shown for the canonical OPLS dataset with a cutoff radius of 1.55 nm.

## S6.4 Comparison of the experimental and calculated $p(\sigma)$

We compared the normalized distributions $p(\sigma)$ calculated from the generated ensembles with the experimental distribution of the model compound **1** in the fully deuterated solvent (Sample **1** in the main text). Upon the comparison, we introduced a cutoff radius $r_{\text{cut}}$ which models the protons that are excluded from the spectral diffusion and, thus, are not observable in ih-RIDME. The cutoff radius was scanned in the range of 0.5-2.0 nm. Since the generated ensembles in four different models are close, the calculated distributions are also close and we discuss them together.

The comparison with the experiment was based on the calculation of rmsd, mean, width and skewness of the distributions (Figure S15). The rmsd criterion and the comparison of the mean values suggested that the best agreement with the experiment is achieved at $r_{\text{cut}} = 1.55\ \text{nm}$. The calculated distributions with $r_{\text{cut}} = 1.55\ \text{nm}$ are narrower than the experimental result (panel C in Figure S15). This may be related to the overestimation of the distribution width from the ih-RIDME fitting. In addition, the experimental distribution is characterized by a stronger skewness than those calculated. These findings may also indicate that the unrestrained conformational ensemble of the model compound **1** is not an accurate enough model. In this scenario, the ih-RIDME data would be useful to refine the confirmation ensemble.

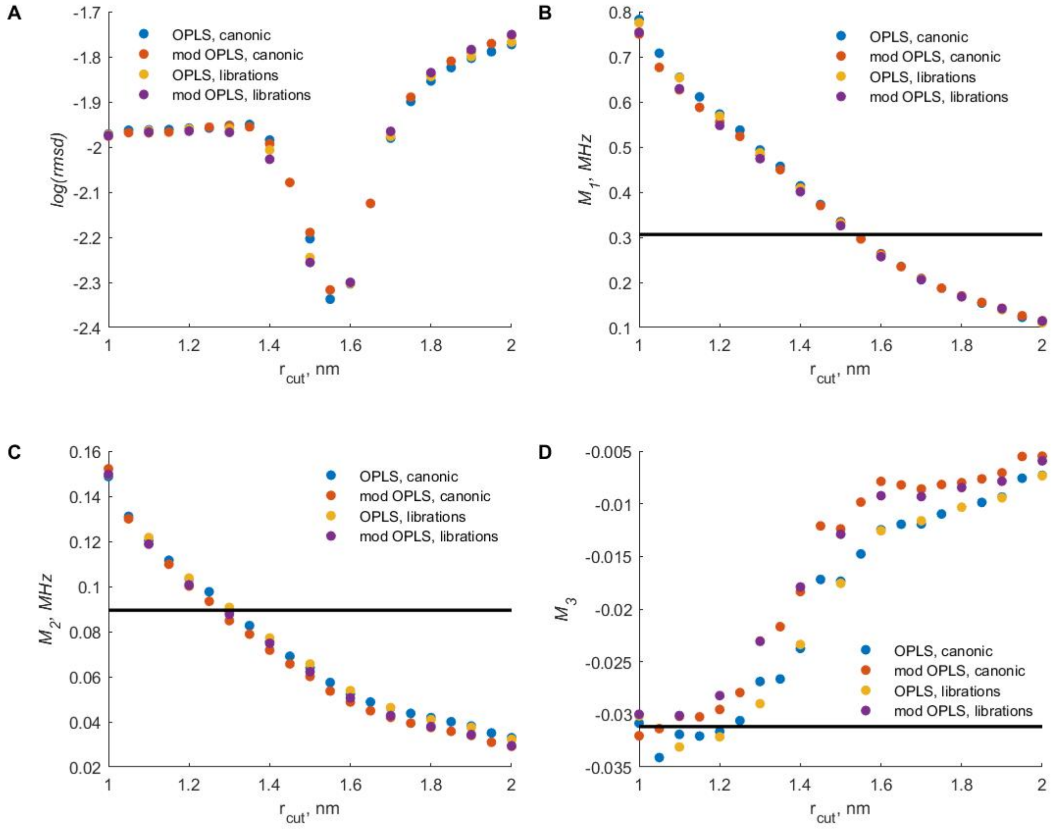


**Figure S15.** A. The rmsd for local proton density from experimental data and calculated from datasets constructed with various OPLS profiles at different cutoff radii. The mean ($M_1 = \mu_1$, panel B), the width ($M_2 = \mu_2^{1/2}$, panel C), and the skewness ($M_3 = \mu_3/\mu_2^{3/2}$, panel D) of local proton density distribution, calculated from experimental RIDME traces (black line), and for datasets constructed with various OPLS profiles at different cutoff radius (coloured circles). All parameters were estimated in the range of $\sigma$-axis $0 - 0.48$ MHz.

## S6.5 Ensemble representation with blocking radius

The molecular ensemble of model compound **1** with the optimal blocking radius of 1.55 nm represented by differently coloured protons is presented in Figure S16.

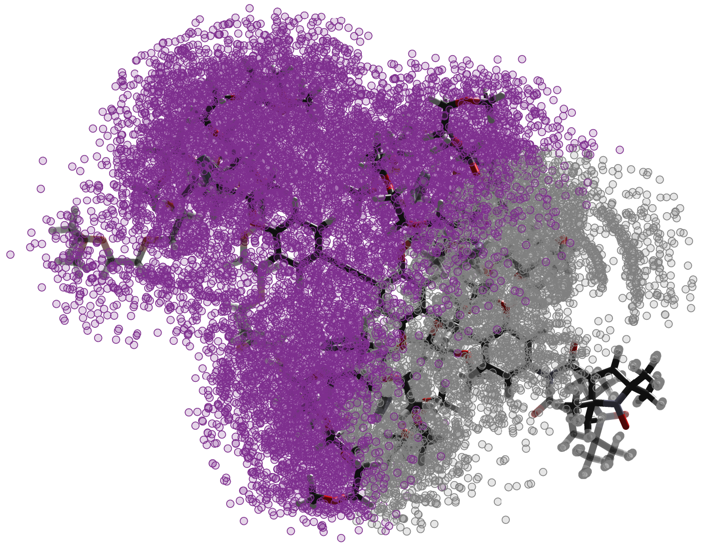

**Figure S16.** 3D structure and ensemble of 200 randomly selected conformers of model compound **1** aligned by the rigid backbone. Magenta circles represent the positions of protons. The protons within the 1.55-nm distance range to the unpaired electron are coloured in grey.

## S7 ih-RIDME sequences

### S7.1 Remote detection in 3p-RIDME

A direct comparison of 3p-RIDME and 3pRD-RIDME (three-pulse with remote detection) traces in Figure S17 shows that the remote detection does not introduce data distortion to the 3p-RIDME data.

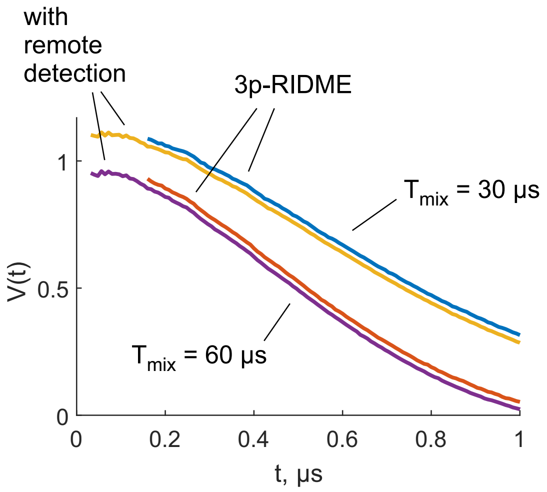


**Figure S17.** Comparison of 3p-RIDME traces with those recorded using the remote detection block (3pRD-RIDME). The latter traces have lower dead time (32 ns) and their shape perfectly overlaps with 3p-RIDME traces demonstrating that remote detection does not introduce data distortion. The measurements were done for TEMPO in $H_2O/H_8$-glycerol at 50 K at Q band.

### S7.2 ESEEM averaging in 4p-RIDME

The ESEEM averaging in 4p-RIDME can be achieved by incrementing the length of delay $d_1$. In the example in Figure S18, the $^2$H-ESEEM in Q band ($1/\nu_I(^2\mathrm{H}) \approx 123$ ns) was averaged by stepping $d_1$ in 16 ns 8 times.

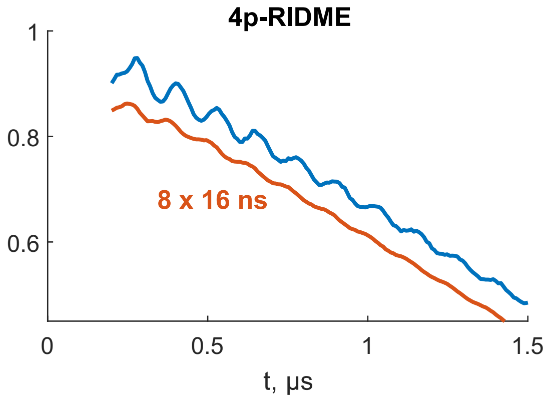


**Figure S18.** 4p-RIDME traces of TEMPO in partially deuterated water/glycerol matrix at Q band. Blue line: without $^2$H-ESEEM averaging protocol. Orange line: with averaging protocol ($d_1$ delay is incremented 8 times with a step of 16 ns). The orange curve is shifted vertically downwards for better visibility.

## S7.3 Comparison of the longitudinal factors

The 5p-RIDME, 3pRD-RIDME and 5pVT-RIDME traces were measured at Q band with a solution of TEMPO radical in water/glycerol solvent (protonation degree 51%). The trace shapes differ in the time domain (see Figure 10 in the main text). After the division by the reference trace, the datasets show a good extent of similarity. In a homogeneous solution, the trace division eliminates the transverse factor in the ih-RIDME traces. The good shape match of the divided traces means that the longitudinal factors in the three versions of the HYSDEMON experiment are very close and the differences in the undivided data are determined by the transverse factors. This confirms the signal model for an ih-RIDME (HYSDEMON) experiment.

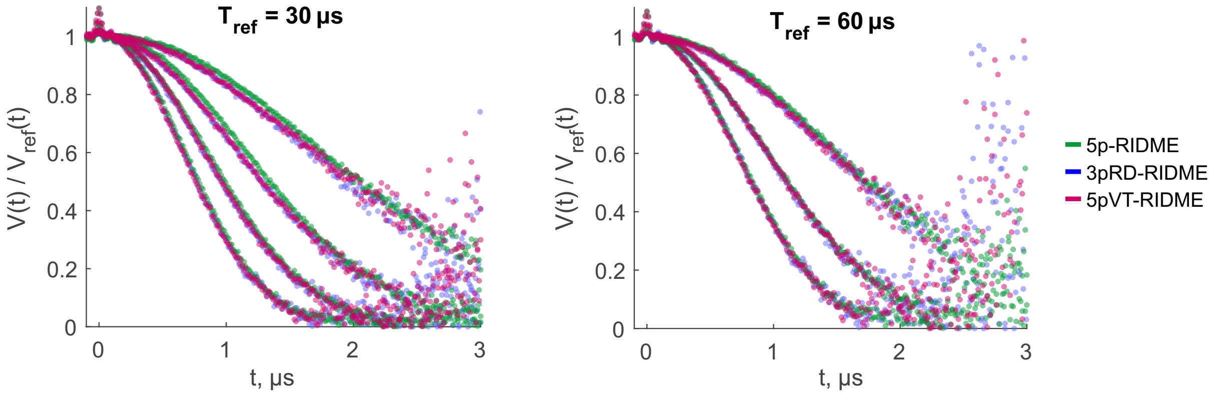


**Figure S19.** Series of ih-RIDME traces (5p-RIDME - green, 3pRD-RIDME - blue, 5pVT-RIDME - magenta) divided by a reference trace with $T_{\text{mix}} = 30\ \mu s$ (left panel) and 60 $\mu s$ (right panel).

## S7.4 Extraction of transverse factors $F(t)$

The transverse factors in 3p-RIDME and 5pVT-RIDME traces ($F_{3p}(t)$, respectively, $F_{5p}(t)$; see Figure 11 in the main text) were extracted using the relation

$$F_{\bullet}(t) = \frac{V_{\bullet}(t;T_{\text{mix}})}{R(t;T_{\text{mix}})} \tag{S13}$$

where $V_{\bullet}(t;T_{\text{mix}})$ are the experimental traces (the bullet sign replaces $3p$ or $5p$) at different mixing times and $R(t;T_{\text{mix}})$ are longitudinal factors computed according to (see Eqs. (S3)-(S5))

$$R(t;T_{\text{mix}}) = \boldsymbol{c}_0{}^T \exp(-i\hat{R}\sigma t) \exp\left(\frac{D}{\sigma^3}\hat{\Gamma}T_{\text{mix}}\right) \exp(i\hat{R}\sigma t)\boldsymbol{c}_0\,. \tag{S14}$$

The parameters for simulation were taken from Ref. Kuzin et al. (2022) and were $D/\sigma^3 = 18\ \text{ms}^{-1}$ and $\sigma = 1.23$ MHz.

## References


Esquivel, R. O., Liu, S., Angulo, J. C., Dehesa, J. S., Antolín, J., and Molina-Espíritu, M.: Fisher information and steric effect: Study of the internal rotation barrier of ethane, J. Phys. Chem. A, 115, 4406–4415, 2011.

Halgren, T. A.: MMFF VI. MMFF94s option for energy minimization studies, J. Comput. Chem., 20, 720–729, 1999.

Hanwell, M. D., Curtis, D. E., Lonie, D. C., Vandermeersch, T., Zurek, E., and Hutchison, G. R.: Avogadro: an advanced semantic chemical editor, visualization, and analysis platform, J. Cheminf., 4, 1–17, 2012.

Hoffmann, M. M., Too, M. D., Paddock, N. A., Horstmann, R., Kloth, S., Vogel, M., and Buntkowsky, G.: On the behavior of the ethylene glycol components of polydisperse polyethylene glycol PEG200, J. Phys. Chem. B, 127, 1178–1196, 2023.

Keller, K., Doll, A., Qi, M., Godt, A., Jeschke, G., and Yulikov, M.: Averaging of Nuclear Modulation Artefacts in RIDME Experiments, J. Magn. Reson., 272, 108–113, 2016.

Kuzin, S., Jeschke, G., and Yulikov, M.: Diffusion equation for the longitudinal spectral diffusion: the case of the RIDME experiment, Phys. Chem. Chem. Phys., 24, 23 517–23 531, 2022.

Ritsch, I., Hintz, H., Jeschke, G., Godt, A., and Yulikov, M.: Improving the accuracy of Cu (II)–nitroxide RIDME in the presence of orientation correlation in water-soluble Cu (II)–nitroxide rulers, Phys. Chem. Chem. Phys., 21, 9810–9830, 2019.
